# NMR Discrimination of d- and l-α-Amino Acids at Submicromolar Concentration via Parahydrogen-Induced Hyperpolarization

**DOI:** 10.1021/jacs.2c11285

**Published:** 2023-01-10

**Authors:** Lennart Dreisewerd, Ruud L. E. G. Aspers, Martin C. Feiters, Floris P. J. T. Rutjes, Marco Tessari

**Affiliations:** Institute for Molecules and Materials, Radboud University, Heyendaalseweg 135, 6525AJ Nijmegen, The Netherlands

## Abstract

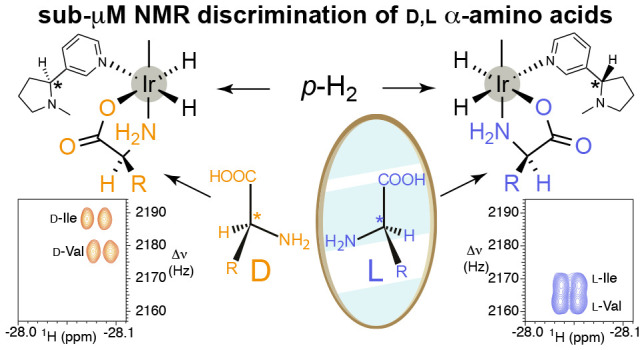

Differentiation of enantiomers represents an important research area for pharmaceutical, chemical, and food industries. However, enantiomer separation is a laborious task that demands complex analytical techniques, specialized equipment, and expert personnel. In this respect, discrimination and quantification of d- and l-α-amino acids is no exception, generally requiring extensive sample manipulation, including isolation, functionalization, and chiral separation. This complex sample treatment results in high time costs and potential biases in the quantitative determination. Here, we present an approach based on the combination of non-hydrogenative parahydrogen-induced hyperpolarization and nuclear magnetic resonance that allows detection, discrimination, and quantification of d- and l-α-amino acids in complex mixtures such as biofluids and food extracts down to submicromolar concentrations. Importantly, this method can be directly applied to the system under investigation without any prior isolation, fractionation, or functionalization step.

Once considered biologically irrelevant, the interest in d-α-amino acids (D-AAs) has significantly grown over the past decades. The realization that important niche roles in biological systems are fulfilled by D-AAs^1,2^ is largely due to the development of analytical methodologies with adequate enantiodiscrimination. One of the reasons for the growing interest in the separation and quantitative determination of D-AAs lies in clinical diagnostics. Abnormal levels of D-AAs in human tissues and biofluids have been related to diseases, such as chronic renal failure,^3^ liver dysfunction,^4^ schizophrenia,^1,5^ and Alzheimer’s.^6^ In addition, an important area of interest is represented by the analysis of D-AAs content in food as it can provide information about its quality, authenticity, or microbial contamination.^7^

Despite the development of enantioselective techniques, discrimination of enantiomers remains a laborious task demanding complex analytical methods.^8^ This issue is further aggravated when dealing with complex mixtures, for which isolation, derivatization, and chiral separation by chromatography or capillary electrophoresis are generally required. In addition to the time costs, this extensive sample manipulation can result in potential biases in the quantitative determination.

Here, we present an approach that requires virtually no sample treatment to achieve discrimination of amino acid enantiomers via hyperpolarized NMR spectroscopy. NMR is widely used to obtain chiral information, provided suitable derivatizing or solvating agents are used to produce diastereomeric complexes for enantiomer resolution.^8−10^ The method we propose achieves differentiation of α-amino acid enantiomers through their noncovalent interactions with a chiral iridium–heterocyclic carbene catalyst. This approach is conceptually similar to the ^19^F NMR chemosensory system based on a ligand–metal complex, recently reported for enantiomeric discrimination of chiral amines.^11^ Over the past decade, the iridium catalyst employed in this work has been widely used to achieve nuclear spin hyperpolarization on different classes of ligands,^12−19^ with techniques such as signal enhancement by reversible exchange (SABRE)^12,20−24^ and high field non-hydrogenative parahydrogen-induced polarization (nhPHIP).^25−27^

As sketched in Figure 1, a α-amino acid associates with the iridium catalyst in a bidentate fashion, involving both the amino and the carboxyl groups.^19^ While this tight binding prevents amino acid dissociation from the metal, parahydrogen (*p-*H_2_) and the cosubstrate (cosub), a ligand added *ad hoc* to the solution,^20^ associate reversibly to the remaining equatorial sites of the catalyst. Formation at high magnetic field of this asymmetric complex allows the conversion of the singlet order of *p-*H_2_ to hydride magnetization that is enhanced up to 1000 times compared to thermal equilibrium.^25,28,29^ The ligands dissociation/association determines a continuous refreshment of *p-*H_2_ in the complex. Consequently, fast sample repolarization can be achieved by bubbling *p-*H_2_ through the solution, which allows the acquisition of multiscan, multidimensional hyperpolarized NMR spectra.^30^

**Figure 1:**
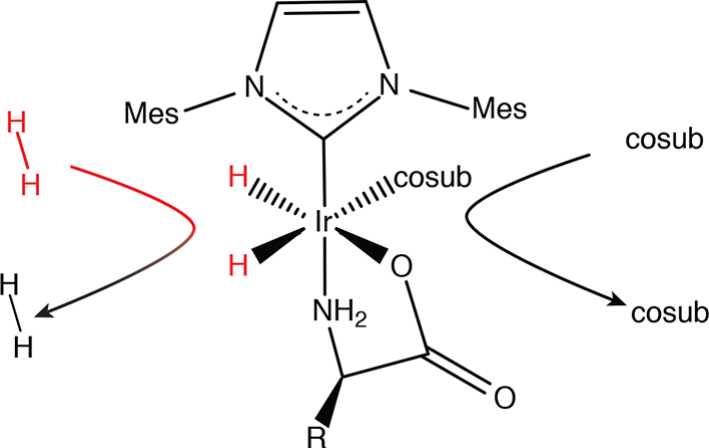
Transient complex formed upon association of a α-amino acid, *p-*H_2_, and an additional ligand (cosub) to an iridium–heterocyclic carbene catalyst.

Because of this sensitivity increase, measurement of the hydrides at submicromolar concentrations is possible, well below typical NMR limits of detection. Notably, the ligand dissociation is slow on the NMR time scale, resulting in long-living complexes and, consequently, sharp hydride signals. For each α-amino acid, association with the iridium catalyst is signaled by a set of hydride resonances at well-defined chemical shifts. These hyperpolarized hydrides can, therefore, act as probes to indirectly reveal the presence of specific amino acids in solution.^19^ Importantly, since hydrides resonate well below −20 ppm, a region of the ^1^H spectrum that is generally empty, they do not overlap with the signals originating from the sample matrix. Submicromolar sensitivity and the absence of matrix spectral background render the nhPHIP-NMR technique highly suitable for the detection of α-amino acids in complex mixtures, with minimal sample treatment requirements.^19^

The combination of the chiral center at the Cα of an l-α-amino acid with the stereogenic center on iridium in the catalyst leads to the formation of two diastereomeric complexes that can interconvert via dissociation/association of the ligands in the equatorial plane (i.e., the cosubstrate or the carboxyl group of the amino acid).^19^ For each complex, a pair of hydride resonances is observed at approximately −23 and −28 ppm, respectively. When using an achiral molecule such as pyridine as cosubstrate, the complexes of the d- and l-enantiomers of an α-amino acid are enantiomers too, giving rise to hydride signals resonating at identical frequencies. This is illustrated in Figure 2A, displaying the high field hydride resonances measured for a racemic mixture of alanine with pyridine as cosubstrate. The hydride signals produced by the d- and l-enantiomers of alanine can be resolved by using a chiral, enantiomerically pure, pyridine derivative, such as (*S*)-nicotine, as cosubstrate (see the Supporting Information). In this case, in fact, the complexes formed by the d- and l-α-amino acids are diastereoisomers, giving rise to four sets of signals, as displayed in Figure 2B for the same racemic mixture of alanine.

**Figure 2:**
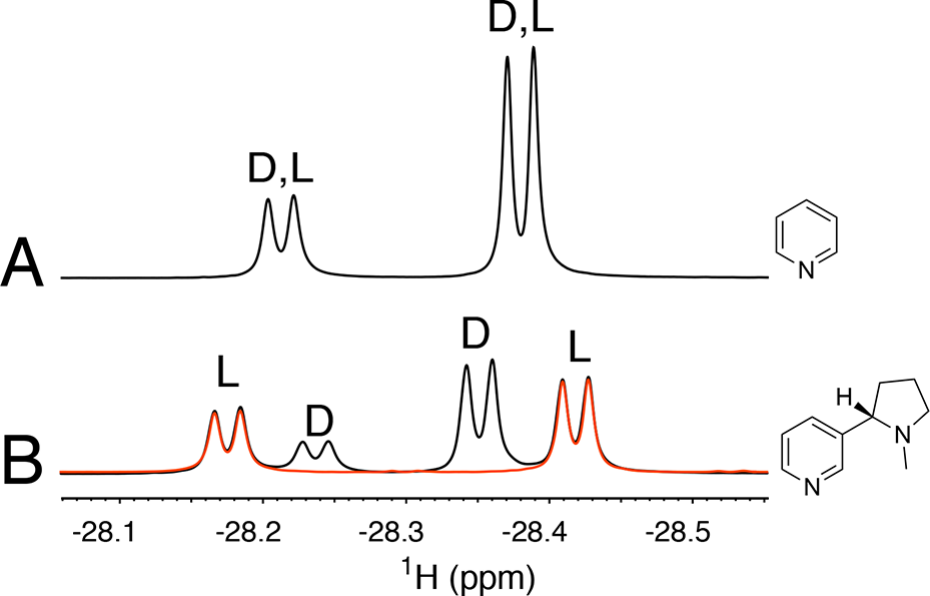
nhPHIP-NMR signals of the high field hydrides for a racemic mixture of alanine (200 μM) in CH_3_OH:H_2_O 95:5 (v/v) using pyridine (A) or (*S*)-nicotine (B) as cosubstrate. Both spectra were acquired at 10 °C and 500 MHz ^1^H resonance frequency in the presence of 0.4 mM Ir-IMes catalyst, 7.2 mM cosubstrate, and 5 bar of 51%-enriched *p-*H_2_. The structures of the cosubstrates are reported. The spectrum in red displays the hydride signals of the l-enantiomer of alanine in the presence of (*S*)-nicotine.

Note that changing the chirality of the cosubstrate, i.e., using (*R*)- instead of (*S*)-nicotine, produces a spectrum identical with the one in Figure 2B, but with the signal assignment for the d- and l-enantiomers being swapped (see the Supporting Information).

Similar resonance patterns are observed for the racemates of all natural α-amino acids, with the exception of histidine, methionine, and cysteine, which seem to adopt a different binding mode with the catalyst, possibly involving the side chain. When dealing with signal crowding, a 2D nhPHIP zero quantum experiment can be used to resolve the resonances of the hydrides,^31,32^ as shown in Figure 3 for a racemic mixture of 16 α-amino acids and glycine, with most resonances accessible for a quantitative analysis. Typically, the high field signals are consulted for quantification, as these are better resolved than their low field counterparts.

**Figure 3:**
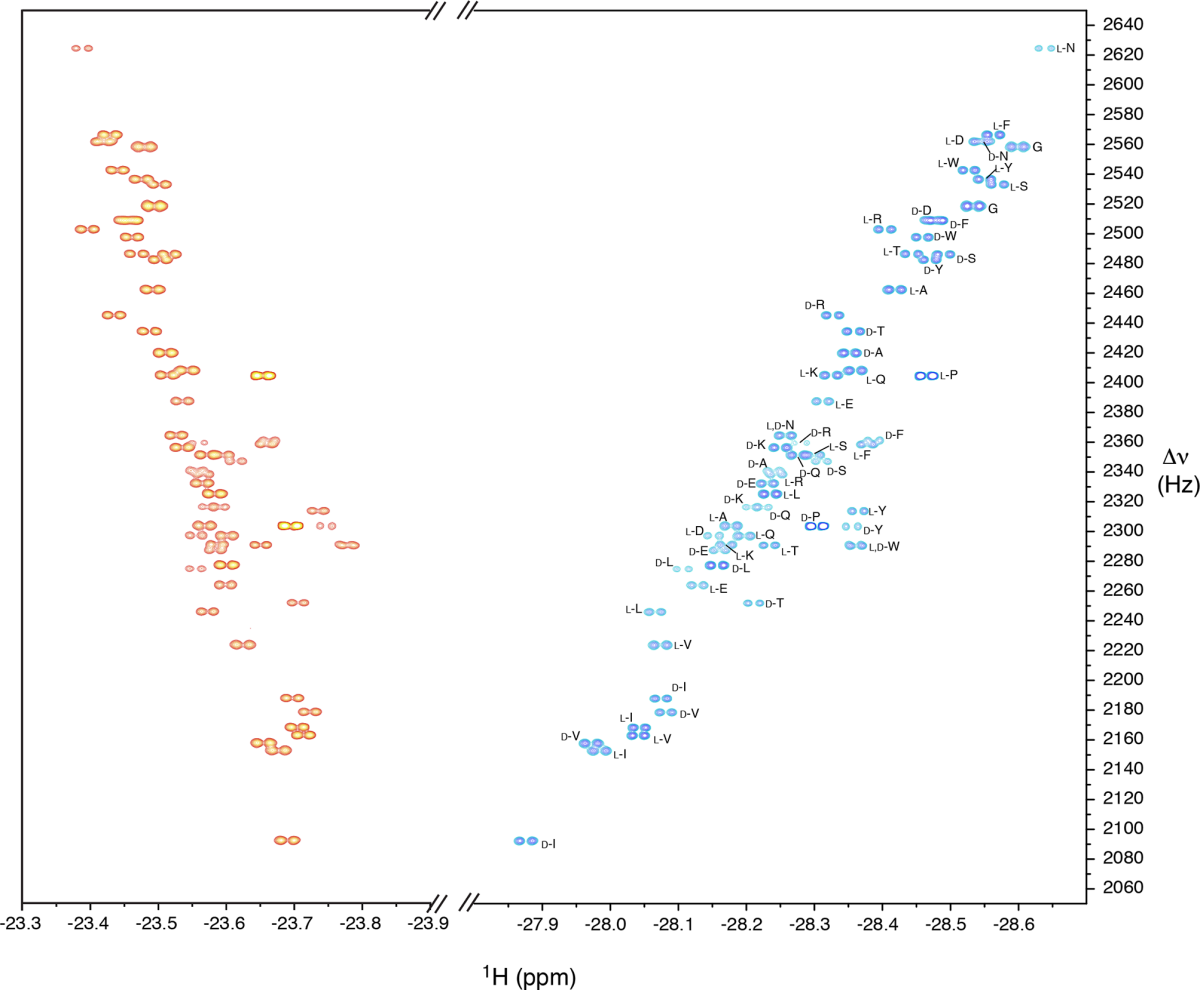
2D nhPHIP zero quantum spectrum of a racemic mixture of 16 α-amino acids (10 μM each enantiomer) in CH_3_OH:H_2_O 95:5 (v/v). The spectrum was acquired at 10 °C and 500 MHz ^1^H resonance frequency. The signal assignment is indicated.

We have previously demonstrated that the integrals of the nhPHIP-NMR signals depend linearly on the concentrations of α-amino acids in solution.^19^ It is therefore possible to calculate the enantiomeric ratio from such integrals, provided the difference between nhPHIP enhancement for the d- and l-amino acid complexes is taken into account. This can be realized by measuring the integral ratio (*R*_rac_) between two L- and D-nhPHIP-NMR signals for a racemic solution (see the Supporting Information).

This value can then be used to determine the enantiomeric ratio (*E*_r_) for a generic sample measured under the same experimental conditions (e.g., magnetic field strength, temperature, and solvent composition) according to this expression
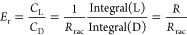
where *C*_L_ and *C*_D_ refer to the analytical concentration of the l- and d-enantiomer, respectively, and *R* denotes the L/D integral ratio obtained using the same two resonances previously used to determine *R*_rac_.

The nhPHIP-NMR approach was applied to a methanolic extract of instant coffee, for which 24 signals from α-amino acids enantiomers were assigned. The presence of d-α-amino acids in coffee results from the racemization of the l-enantiomers during the roasting process.^33−35^ Note that sample preparation solely involved mixing the methanolic coffee extract with the iridium/(*S*)-nicotine solution, without any additional treatment before the nhPHIP-NMR measurement. The enantiomeric ratio was determined for five amino acids, as summarized in Table 1.

**Table 1:** Integral and L-/D-Enantiomeric Ratios for Five α-Amino Acids in Instant Coffee

AA	L (ppm)	D (ppm)	*R*	*R*_rac_	*E*_r_
Ala	–28.424	–28.356	1.84^a^ (0.02)	1.00	1.84 (0.02)
Ile	–27.99	–27.875	4.41	1.09	4.05
Leu	–28.238	–28.158	2.9^a^ (0.1)	1.05	2.8 (0.1)
Pro	–28.471	–28.303	4.5^a^ (0.1)	0.975	4.6 (0.1)
Val	–28.047	–27.794	4.17	0.813	5.13

a Average over three measurements (original sample and coffee spiked twice with dl-Ile and dl-Val); SD in parentheses.

The absolute concentration of amino acid enantiomers can be determined by single-point standard addition, as illustrated below for Ile and Val in the coffee extract. Figure 4 shows the superposition of a region of the 2D spectrum of the original sample (green peaks) and of a second sample spiked with Ile and Val racemates (orange peaks).

**Figure 4:**
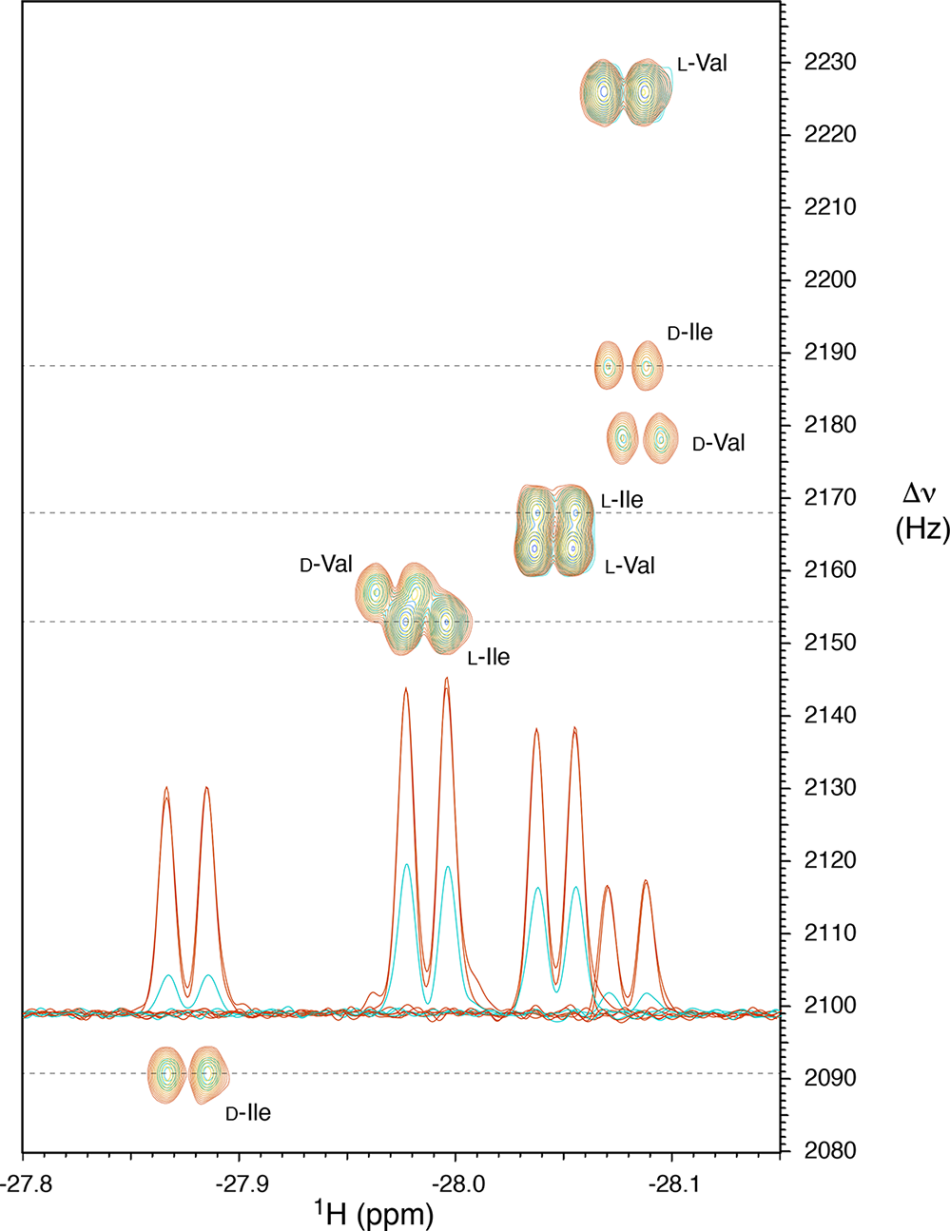
Overlay of a portion of the 2D nhPHIP zero quantum spectra of an extract of instant coffee (green) and of a sample spiked with 5.1 μM dl-Ile and 5.1 μM dl-Val (orange). Each 2D experiment was acquired in CH_3_OH:H_2_O 95:5 (v/v) at 5 °C and 500 MHz ^1^H resonance frequency. The signal assignment is indicated. The 1D traces of the 2D peaks of Ile are displayed for the original mixture (cyan) and for two spiked samples (red).

The absolute concentration of each enantiomer in the coffee extract can be calculated from the values of the enantiomeric ratio before and after spiking as
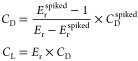


Table 2 summarizes the results of the single-point spiking experiments on Ile and Val. These concentrations correspond to a few mg/kg α-amino acids in roasted coffee, in agreement with data reported in the literature.^35^ Note that these values were calculated from two spiking experiments producing identical results, as evidenced by the 1D traces in Figure 4 (red) that are virtually indistinguishable. However, the precision of these measurements is determined primarily by the signal/noise ratio for the d- and l-complexes in the nonspiked sample of coffee extract. Therefore, on the basis of a signal/noise ratio of 15–20 for d-Ile and d-Val, a conservative estimate of the precision (ca. 10%) was reported in Table 2.

**Table 2:** Concentrations of dl-Ile and dl-Val in Coffee Extract from Single-Point Standard Addition

AA	*C*^spiked^ (μM)	*R*_spiked_	*E*_r_^spiked^	*C* (μM)
d-Ile	2.55	1.637	1.504	0.5 (0.05)
l-Ile	2.55	1.637	1.504	2.0 (0.2)
d-Val	2.55	1.597	1.964	0.78 (0.06)
l-Val	2.55	1.597	1.964	4.0 (0.3)

Discrimination of amino acid enantiomers was further tested on human urine, an area that is receiving increasing attention, particularly as a tool for screening for chronic kidney diseases.^36^ Urine is a challenging type of specimen because of the complexity of its composition^37^ and the presence of concentrated metabolic byproducts that act as Ir–catalyst ligands. As previously demonstrated, a 20-fold dilution of urine in methanol without further sample treatments allows detection of α-amino acids via nhPHIP-NMR, preventing the competition between the cosubstrate and sample innate ligands.^19^

Unlike l-amino acids, mostly reabsorbed by kidney tubules, relatively large portions of d-Ser and d-Ala are normally excreted into urine.^36^ The enantiomeric ratio determined via nhPHIP-NMR on a sample from a healthy volunteer was found in good agreement with the values reported in the literature for both amino acids,^38^ as summarized in Table 3.

**Table 3:** Integral and Enantiomeric Ratios for Ala and Ser in Human Urine

AA	L (ppm)	D (ppm)	*R*	*R*_rac_	*E*_r_	% D
Ala	–28.419	–28.352	2.71	0.911	2.98	25.1 (10.7–28)^a^
Ser	–28.570	–28.490	1.21	0.891	1.36	42.3 (32.6–56.2)^a^

a From ref (38).

Note that a different value of *R*_rac_ was used for alanine in the analysis of coffee extract due to different measuring temperatures (10 °C for urine and 5 °C for instant coffee).

The method we have proposed requires the association of a homochiral cosubstrate with the iridium catalyst to resolve the nhPHIP–NMR signals of d- and l-amino acid complexes. Note, however, that these roles could be reversed by employing a homochiral amino acid to discriminate the enantiomers of a generic chiral ligand (such as nicotine). Therefore, enantiomers discrimination by nhPHIP-NMR can be extended to other classes of chiral compounds that associate with the iridium catalyst together with an enantiomerically pure cosubstrate and *p-*H_2_.

In conclusion, we have presented a method to discriminate and quantify α-amino acids enantiomers in solution. This approach was demonstrated for all proteinogenic amino acids, except histidine, methionine, and cysteine that seem to adopt different binding modes to the iridium catalyst. Because of the sensitivity increase determined by hyperpolarization and the absence of background signals from the sample matrix, this approach is ideally suited for complex mixtures, such as natural extracts, food, and biofluids, down to submicromolar α-amino acid concentrations. Mixtures of d- and l-α-amino acids can be resolved with a 2D nhPHIP zero quantum spectrum that provides global and quantitative information about the different amino acid enantiomers in solution, without resorting to chromatographic fractionation. Analogous to recently proposed NMR approaches based on chiral solvating agents^39,40^ this nhPHIP NMR method allows direct enantiospecific detection of amino acids in complex mixtures, with the additional advantage of submicromolar sensitivity. Importantly, avoiding involved, multistep analytical protocols allows a quantitative determination of d-enantiomers with negligible bias, which can assist the quantification process by other techniques as in the recently proposed NMR-guided MS quantitation approach.^41^

## Supporting Information Available

Experimental details including a description of the experimental instrumentation, the NMR experiments, data analysis, protocols for coffee and urine samples preparation, high-field region of the 1D nhPHIP-NMR spectra for 16 dl-α-amino acids, assignment table for amino acids enantiomers, structure and nomenclature of the amino acids diastereoisomeric complexes, determination of the enantiomeric ratio, determination of the integral ratio for enantiomers in different matrices at known concentrations. (PDF)

## References

[ref1] Labrie V.; Roder J. C. The involvement of the NMDA receptor D-serine/glycine site in the pathophysiology and treatment of schizophrenia. Neurosci. Biobehav. Rev. 2010, 34, 351–372. 10.1016/j.neubiorev.2009.08.002. 19695284

[ref2] Topo E.; Soricelli A.; D’Aniello A.; Ronsini S.; D’Aniello G. The role and molecular mechanism of D-aspartic acid in the release and synthesis of LH and testosterone in humans and rats. Reprod. Biol. Endocrinol. 2009, 7, 120–130. 10.1186/1477-7827-7-120. 19860889 PMC2774316

[ref3] Young G. A.; Kendall S.; Brownjohn A. M. D-Amino acids in chronic renal failure and the effects of dialysis and urinary losses. Amino Acids 1994, 6, 283–293. 10.1007/BF00813748. 24189736

[ref4] Nagata Y.; Masui R.; Akino T. The presence of free D-serine, D-alanine and D-proline in human plasma. Experientia 1992, 48, 986–988. 10.1007/BF01919147. 1426150

[ref5] Kumashiro S.; Hashimoto A.; Nishikawa T. Free D-serine in post-mortem brains and spinal cords of individuals with and without neuropsychiatric diseases. Brain Res. 1995, 681, 117–125. 10.1016/0006-8993(95)00307-C. 7552268

[ref6] Fisher G. H.; D’Aniello A.; Vetere A.; Padula L.; Cusano G. P.; Man E. H. Free D-aspartate and D-alanine in normal and Alzheimer brain. Brain Res. Bull. 1991, 26, 983–985. 10.1016/0361-9230(91)90266-M. 1933416

[ref7] Marcone G. L.; Rosini E.; Crespi E.; Pollegioni L. D-amino acids in foods. Appl. Microbiol. Biotechnol. 2020, 104, 555–574. 10.1007/s00253-019-10264-9. 31832715

[ref8] aDifferentiation of Enantiomers I; Schurig V., Ed.; Springer: Heidelberg, 2013.

[ref9] Sau S. P.; Ramanathan K. V. Visualization of Enantiomers in the Liquid-Crystalline Phase of a Fragmented DNA Solution. J. Phys. Chem. B 2009, 113, 1530–1532. 10.1021/jp806534v. 19133792

[ref10] Lesot P.; Reddy U. V.; Suryaprakash N. Exploring the spectral enantiodiscrimination potential of a DNA-based orienting medium using deuterium NMR spectroscopy. Chem. Commun. 2011, 47, 11736–11738. 10.1039/c1cc15097a. 21952675

[ref11] Zhao Y.; Swager T. M. Simultaneous Chirality Sensing of Multiple Amines by ^19^F NMR. J. Am. Chem. Soc. 2015, 137, 3221–3224. 10.1021/jacs.5b00556. 25723526 PMC5818995

[ref12] Adams R. W.; Aguilar J. A.; Atkinson K. D.; Cowley M. J.; Elliott P. I. P.; Duckett S. B.; Green G. G. R.; Khazal I. G.; Lopez-Serrano J.; Williamson D. C. Reversible Interactions with para-Hydrogen Enhance NMR Sensitivity by Polarization Transfer. Science 2009, 323, 1708–1711. 10.1126/science.1168877. 19325111

[ref13] Shchepin R. V.; Barskiy D. A.; Coffey A. M.; Goodson B. M.; Chekmenev E. Y. NMR Signal Amplification by Reversible Exchange of Sulfur-Heterocyclic Compounds Found in Petroleum. ChemistrySelect 2016, 1, 2552–2555. 10.1002/slct.201600761. 27500206 PMC4972496

[ref14] Mewis R. E.; Green R. A.; Cockett M. C. R.; Cowley M. J.; Duckett S. B.; Green G. G. R.; John R. O.; Rayner P. J.; Williamson D. C. Strategies for the Hyperpolarization of Acetonitrile and Related Ligands by SABRE. J. Phys. Chem. B 2015, 119, 1416–1424. 10.1021/jp511492q. 25539423 PMC4315046

[ref15] Iali W.; Rayner P. J.; Alshehri A.; Holmes A. J.; Ruddlesden A. J.; Duckett S. B. Direct and indirect hyperpolarisation of amines using parahydrogen. Chem. Sci. 2018, 9, 3677–3684. 10.1039/C8SC00526E. 29780498 PMC5935062

[ref16] Logan A. W. J.; Theis T.; Colell J. F. P.; Warren W. S.; Malcolmson S. J. Hyperpolarization of Nitrogen-15 Schiff Bases by Reversible Exchange Catalysis with para-Hydrogen. Chem. – Eur. J. 2016, 22, 10777–10781. 10.1002/chem.201602393. 27218241

[ref17] Theis T.; Ortiz G. X.; Logan A. W. J.; Claytor K. E.; Feng Y.; Huhn W. P.; Blum V.; Malcolmson S. J.; Chekmenev E. Y.; Wang Q.; Warren W. S. Direct and cost-efficient hyperpolarization of long-lived nuclear spin states on universal ^15^N_2_-diazirine molecular tags. Sci. Adv. 2016, 2, e1501438 10.1126/sciadv.1501438. 27051867 PMC4820385

[ref18] Gemeinhardt M. E.; Limbach M. N.; Gebhardt T. R.; Eriksson C. W.; Eriksson S. L.; Lindale J. R.; Goodson E. A.; Warren W. S.; Chekmenev E. Y.; Goodson B. M. “Direct” ^13^C Hyperpolarization of ^13^C-Acetate by MicroTesla NMR Signal Amplification by Reversible Exchange (SABRE). Angew. Chem., Int. Ed. 2020, 59, 2–8. 10.1002/anie.201910506. 31661580

[ref19] Sellies L.; Aspers R. L. E. G.; Feiters M. C.; Rutjes F. P. J. T.; Tessari M. Parahydrogen Hyperpolarization Allows Direct NMR Detection of α-Amino Acids in Complex (Bio)mixtures. Angew. Chem., Int. Ed. 2021, 60, 26954–26959. 10.1002/anie.202109588. PMC9299667 34534406

[ref20] Eshuis N.; Hermkens N.; van Weerdenburg B. J. A.; Feiters M. C.; Rutjes F. P. J. T.; Wijmenga S. S.; Tessari M. Toward Nanomolar Detection by NMR Through SABRE Hyperpolarization. J. Am. Chem. Soc. 2014, 136, 2695–2698. 10.1021/ja412994k. 24475903

[ref21] Eshuis N.; van Weerdenburg B. J. A.; Feiters M. C.; Rutjes F. P. J. T.; Wijmenga S. S.; Tessari M. Quantitative Trace Analysis of Complex Mixtures Using SABRE Hyperpolarization. Angew. Chem., Int. Ed. 2015, 54, 1481–1484. 10.1002/anie.201409795. 25469822

[ref22] Theis T.; Truong M. L.; Coffey A. M.; Shchepin R. V.; Waddell K. W.; Shi F.; Goodson B. M.; Warren W. S.; Chekmenev E. Y. Microtesla SABRE Enables 10% Nitrogen-15 Nuclear Spin Polarization. J. Am. Chem. Soc. 2015, 137, 1404–1407. 10.1021/ja512242d. 25583142 PMC4333583

[ref23] Rayner P. J.; Duckett S. B. Signal Amplification by Reversible Exchange (SABRE): From Discovery to Diagnosis. Angew. Chem., Int. Ed. 2018, 57, 6742–6753. 10.1002/anie.201710406. 29316071

[ref24] Barskiy D. A.; Kovtunov K. V.; Koptyug I. V.; He P.; Groome K. A.; Best Q. A.; Shi F.; Goodson B. M.; Shchepin R. V.; Coffey A. M.; Waddell K. W.; Chekmenev E. Y. The Feasibility of Formation and Kinetics of NMR Signal Amplification by Reversible Exchange (SABRE) at High Magnetic Field (9.4 T). J. Am. Chem. Soc. 2014, 136, 3322–3325. 10.1021/ja501052p. 24528143 PMC3985893

[ref25] Wood N. J.; Brannigan J. A.; Duckett S. B.; Heath S. L.; Wagstaff J. Detection of Picomole Amounts of Biological Substrates by para-Hydrogen-Enhanced NMR Methods in Conjunction with a Suitable Receptor Complex. J. Am. Chem. Soc. 2007, 129, 11012–11013. 10.1021/ja074286k. 17711281

[ref26] Hermkens N. K. J.; Eshuis N.; van Weerdenburg B. J. A.; Feiters M. C.; Rutjes F. P. J. T.; Wijmenga S. S.; Tessari M. NMR-Based Chemosensing via p-H_2_ Hyperpolarization: Application to Natural Extracts. Anal. Chem. 2016, 88, 3406–3412. 10.1021/acs.analchem.6b00184. 26901632

[ref27] Reimets N.; Ausmees K.; Vija S.; Reile I. Developing Analytical Applications for Parahydrogen Hyperpolarization: Urinary Elimination Pharmacokinetics of Nicotine. Anal. Chem. 2021, 93, 9480–9485. 10.1021/acs.analchem.1c01281. 34180227

[ref28] Bowers C. R.; Weitekamp D. P. Parahydrogen and Synthesis Allow Dramatically Enhanced Nuclear Alignment. J. Am. Chem. Soc. 1987, 109, 5541–5542. 10.1021/ja00252a049.

[ref29] Natterer J.; Bargon J. Parahydrogen induced polarization. Prog. Nucl. Magn. Reson. Spectrosc. 1997, 31, 293–315. 10.1016/S0079-6565(97)00007-1.

[ref30] Eshuis N.; Aspers R. L. E. G.; van Weerdenburg B. J. A.; Feiters M. C.; Rutjes F. P. J. T.; Wijmenga S. S.; Tessari M. 2D NMR Trace Analysis by Continuous Hyperpolarization at High Magnetic Field. Angew. Chem., Int. Ed. 2015, 54, 14527–14530. 10.1002/anie.201507831. 26437608

[ref31] Sellies L.; Reile I.; Aspers R. L E. G.; Feiters M. C.; Rutjes F. P. J. T.; Tessari M. Parahydrogen induced hyperpolarization provides a tool for NMR metabolomics at nanomolar concentrations. Chem. Commun. 2019, 55, 7235–7238. 10.1039/C9CC02186H. 31165813

[ref32] Fraser R.; Rutjes F. P. J. T.; Feiters M. C.; Tessari M. Analysis of Complex Mixtures by Chemosensing NMR Using para-Hydrogen-Induced Hyperpolarization. Acc. Chem. Res. 2022, 55, 1832–1844. 10.1021/acs.accounts.1c00796. 35709417 PMC9260963

[ref33] Zagon J.; Dehne L. I.; Bögl K. W. D-Amino Acids in Organisms and Food. Nutr. Res. (N. Y.) 1994, 14, 445–463. 10.1016/S0271-5317(05)80182-4.

[ref34] Brückner H.; Hausch M. Detection of free amino acids in food by chiral phase capillary chromatography. J. High Res. Chromatog. 1989, 12, 680–684. 10.1002/jhrc.1240121012.

[ref35] Casal S.; Mendes E.; Oliveira M. B. P. P.; Ferreira M. A. Roast effects on coffee amino acid enantiomers. Food Chem. 2005, 89, 333–340. 10.1016/j.foodchem.2004.02.039.

[ref36] Harada M.; Karakawa S.; Miyano H.; Shimbo K. Simultaneous Analysis of D,L-Amino Acids in Human Urine Using a Chirality-Switchable Biaryl Axial Tag and Liquid Chromatography Electrospray Ionization Tandem Mass Spectrometry. Symmetry 2020, 12, 913–926. 10.3390/sym12060913.

[ref37] Bouatra S.; Aziat F.; Mandal R.; Guo A. C.; Wilson M. R.; Knox C.; Bjorndahl T. C.; Krishnamurthy R.; Saleem F.; Liu P.; Dame Z. T.; Poelzer J.; Huynh J.; Yallou F. S.; Psychogios N.; Dong E.; Bogumil R.; Roehring C.; Wishart D. S. The Human Urine Metabolome. PLoS One 2013, 8, e73076 10.1371/journal.pone.0073076. 24023812 PMC3762851

[ref38] Hesaka A.; Sakai S.; Hamase K.; Ikeda T.; Matsui R.; Mita M.; Horio M.; Isaka Y.; Kimura T. D-Serine reflects kidney function and diseases. Sci. Rep. 2019, 9, 1–8. 10.1038/s41598-019-41608-0. 30911057 PMC6434045

[ref39] Pérez-Trujillo M.; Lindon J. C.; Parella T.; Keun H. C.; Nicholson J. K.; Athersuch T. J. Chiral Metabonomics: ^1^H NMR-Based Enantiospecific Differentiation of Metabolites in Human Urine via Direct Cosolvation with β-Cyclodextrin. Anal. Chem. 2012, 84, 2868–2874. 10.1021/ac203291d. 22320312

[ref40] Kuhn L. T.; Motiram-Corral K.; Athersuch T. J.; Parella T.; Pérez-Trujillo M. Simultaneous Enantiospecific Detection of Multiple Compounds in Mixtures using NMR Spectroscopy. Angew. Chem., Int. Ed. 2020, 59, 23615–23619. 10.1002/anie.202011727. 32959941

[ref41] Nagana Gowda G. A.; Djukovic D.; Bettcher L. F.; Gu H.; Raftery D. NMR-Guided Mass Spectrometry for Absolute Quantitation of Human Blood Metabolites. Anal. Chem. 2018, 90, 2001–2009. 10.1021/acs.analchem.7b04089. 29293320 PMC6260976

