# Supplementary material for: NMR Discrimination of d- and l-α-Amino Acids at Submicromolar Concentration via Parahydrogen-Induced Hyperpolarization
Source: J Am Chem Soc. 2023 Jan 10;145(3):1518–23. doi: 10.1021/jacs.2c11285 (PMC9880991; doi:10.1021/jacs.2c11285)
Supplement: Supplementary file 1 — ja2c11285_si_001.pdf [file ja2c11285_si_001.pdf]

Supporting Information

NMR Discrimination of D- and L-α-Amino Acids at Submicromolar Concentration via Parahydrogen Induced Hyperpolarization

Lennart Dreisewerd, Ruud L. E. G. Aspers, Martin C. Feiters, Floris P. J. T. Rutjes and Marco Tessari*

Institute for Molecules and Materials, Radboud University, Heyendaalseweg 135, 6525AJ Nijmegen, The Netherlands.

Table of contents

Section S1. Chemicals S2

Section S2. Diastereoisomeric Complexes S4

Section S3. NMR experiments S6

Section S4. Resonance assignment of amino acids enantiomers S8

Section S5. Artificial mixture of amino acids racemates S13

Section S6. Enantiomeric ratio from nhPHIP-NMR S15

Section S7. Integral ratio for enantiomers in different matrices S16

Section S8. Application to complex mixtures of known concentrations S18

Section S9. Instant coffee S19

Section S10. Enantiomeric ratio of Ala and Ser in human urine S20

Section S11. References S21

S1 Chemicals

All chemicals were used as supplied. The following chemicals were purchased from Acros Organics BVBA (Order via Fisher Scientific): D-alanine (D-Ala); Fluka AG: DL-alanine (DL – Ala), L-lysine (L-Lys), L-valine (L-Val); Sigma-Aldrich Chemie (Merck Life sciences N.V.): DL-arginine (DL-Arg), DL-asparagine mono-hydrate (DL-Asn), DL-aspartic acid (DL-Asp), DL-glutamic acid monohydrate (DL-Glu), DL-isoleucine (DL-Ile), DL-lysine (DL-Lys), DL-pipecolinic acid, DL-proline (DL-Pro), D-proline (D-Pro), DL-tyrosine (DL-Tyr), piperidine hydrochloride, piperidine, water for HPLC, (*S*)-nicotine); Sigma Chemical Company: L-alanine (L-Ala), L-arginine (L-Arg), L-asparagine (L-Asn), L-aspartic acid (L-Asp), L-glutamic acid monosodium salt (L-GLu), L-glutamine (L-Gln), glycine (Gly), L-isoleucine (L-Ile), L-leucine (L-Leu), L-proline (L-Pro), L-phenylalanine (L-Phe), L-serine (L-Ser), L-threonine (L-Thr), L-tryptophan (L-Trp), L-tyrosine (L-Tyr); ABCR GmbH KG: DL-glutamine (DL- Gln); BdH DL-leucine (DL-Leu); Fisher Scientific: DL-phenylalanine (DL-Phe), DL-threonine (DL-Thr), DL-tryptophan (DL-Trp), DL-valine (DL-Val); Fluorochem EU: L-pipecolinic acid, DL-serine (DL-Ser); LiChrosolv: (methanol, hypergrade for LC-MS). The Ir-complex precursor [Ir(COD)(IMes)Cl](IMes=1,3-bis(2,4,6-trimethylphenyl)imidazole-2-ylidene; COD=cyclooctadiene) was synthesized according to published methods[1].

p-H_2 supply, degassing and pressurizing

The *p*-H_2 supply was ensured via a cryobath consisting of a H_2 vessel (2 L) that is encapsulated in a liquid nitrogen bath (77 K). Inside the vessel 4-8 MESH activated charcoal (Sigma-Aldrich) ensures an effective conversion of H_2 (purity 5.0) to enriched *p*-H_2 (51 %), which can be transferred to a portable cylinder, possessing an adjustable output pressure valve (Nitrous Oxide Systems, Holley Performance Products, Bowling Green, KY, US, aluminium). Degassing and continuous *p*-H_2 saturation of the samples were achieved via an in-house build bubble set-up (Figure S.1). Before the catalyst precursor activation, the sample mixture was saturated with nitrogen (N_2) in order to remove any oxygen (O_2). In the next step, the mixture was saturated with *p*-H_2, resulting in catalyst precursor activation. At the beginning of each 1D nhPHIP-NMR or 2D nhPHIP Zero Quantum transient, the solution was refreshed with *p*-H_2. The refreshment process consists of four steps, whereby initially the NMR-tube pressure is reduced from 5 to 4 Bar via a relief valve that is attached to the so-called vent-line (0.25 s). Secondly, the mixture is re-saturated with *p*-H_2, re-establishing a tube pressure of 5 Bar (0.5-3.0 s). By applying pressure on top of the liquid level, any residual bubbling is suppressed (0.25 s). Lastly, a final delay is applied, allowing stabilization of the sample (0.5 s).

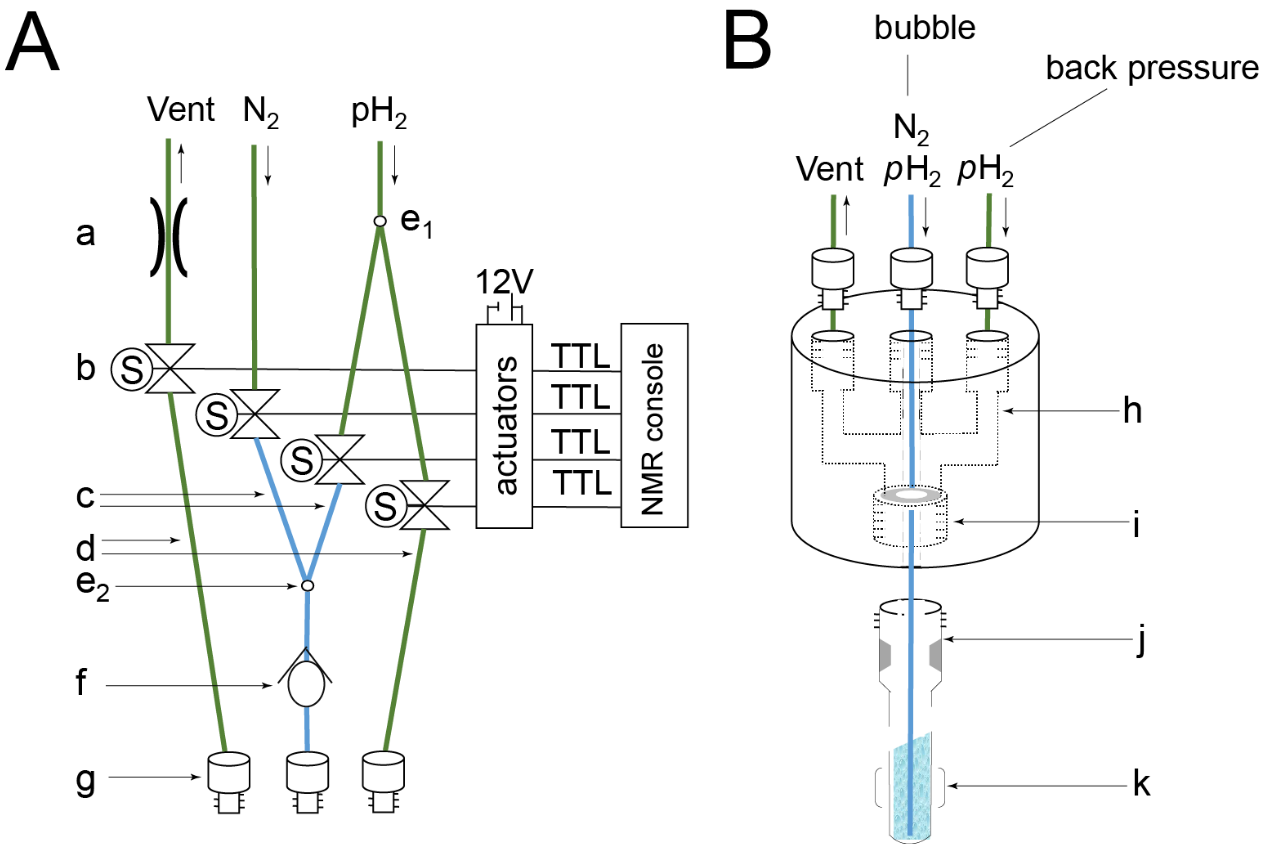

Figure S.1: (**A**) Schematic depiction of the in-house build *p*-H_2 bubbling set-up. **a** an adjustable pressure relief valve calibrated at 4 Bar; **b** solenoid valves controlled via TTL lines powered by an external 12 V source and timed via trigger commands in the pulse sequence taking care of a controlled supply of N_2 and *p*-H_2; **c** 1/16 O.D. PEEK tubing with 0.010 I.D. (blue); **d** 1/16 O.D. PEEK tubing with 0.030 I.D. (green); Y splitter: (**e1**) splitting 5 bar p-H_2 source into a "bubble" and a "backpressure" line, (**e2**) combining 5 Bar N_2 and *p*-H_2 sources into a joined bubble line; **f** one-way valve; **g** tube fitting UNF 10-32 connectors. (**B**) **h** headpiece connecting the PEEK tubing holding the 7 thin wall QPV NMR tube; **i** UNF 7/16-20 thread holding the QPV NMR tube – using a 2 mm silicon disc on top of the tube to close off the system; **j** Wilmad 7 thin wall QPV NMR tube; **k** area of detection with the bubble line centered down to the bottom of the NMR tube.

S2 Diastereoisomeric complexes

A chelating ligand can coordinate in an octahedral complex in two non-superimposable ways, denoted as anti-clockwise "*A*" and clockwise "*C*" (see figure s.2), which are not mirror images. Therefore, when using pyridine as cosubstrate, even in combination with an enantiopure L-amino acid, two diastereoisomeric complexes are obtained, *e.g.* *A*/L and *C*/L. If the amino acid is present as racemate, also the D-enantiomer forms two diastereoisomeric complexes (*A*/D and *C*/D). However, the complexes *A*/D and *C*/L are enantiomers, as well as *C*/D and *A*/L, which explains the presence of only two pairs of hydride signals for the racemate of alanine in the presence of pyridine as cosubstrate.

(*S*)-nicotine is an additional source of chirality for the amino acid complexes. When only the L-amino acid and (*S*)-nicotine are present, one expects two diastereoisomeric complexes: *A*/L/*S* and *C*/L/*S*. However in the presence of an amino acid racemate, also the complexes *A*/D/*S* and *C*/D/*S* are formed, for a total of four diastereoisomers among which no pairs of enantiomers can be found (see figure s.2 below). This is in agreement with the presence of four pairs of hydride signals for the racemate of alanine in the presence of (*S*)-nicotine.

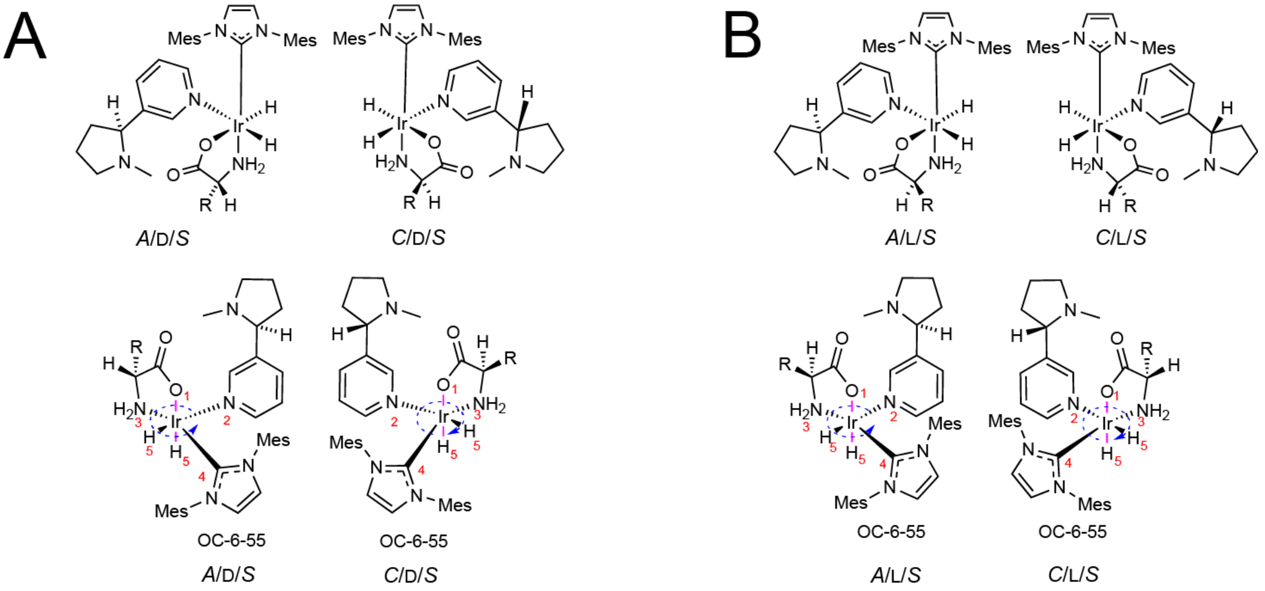

Figure S.2: Top Structures of the $[Ir(H)_2(IMES)(S\text{-Nic})AA]$ complexes formed by (**A**) D- and (**B**) L-α-amino acids (for all natural α-amino acids except cysteine, for which (A) and (B) display L- and D-enantiomers, respectively). **Bottom** Structures of $[Ir(H)_2(IMES)(S\text{-Nic})AA]$ complexes explicitly reporting the priorities of the ligands in the equatorial plane according to Cahn-Ingold-Prelog rules to assign clockwise (*C*) or anti-clockwise (*A*) rotation senses. The IUPAC name of each complex is indicated by: OC-6 (polyhedral symbol), 55 (configuration index) and *A* or *C* (chirality symbol). D or L indicates the relative configuration of the amino acid chiral center and *S* indicates the absolute configuration of nicotine.

When using (*R*)-nicotine instead of (*S*)-nicotine as cosubstrate, an amino acid racemate produces four diastereoisomeric complexes, namely *C*/L/*R*, *C*/D/*R*, *A*/L/*R*, and *A*/D/*R*, that are the respective enantiomers of *A*/D/*S*, *A*/L/*S*, *C*/D/*S*, and *C*/L/*S* formed with (*S*)-nicotine.

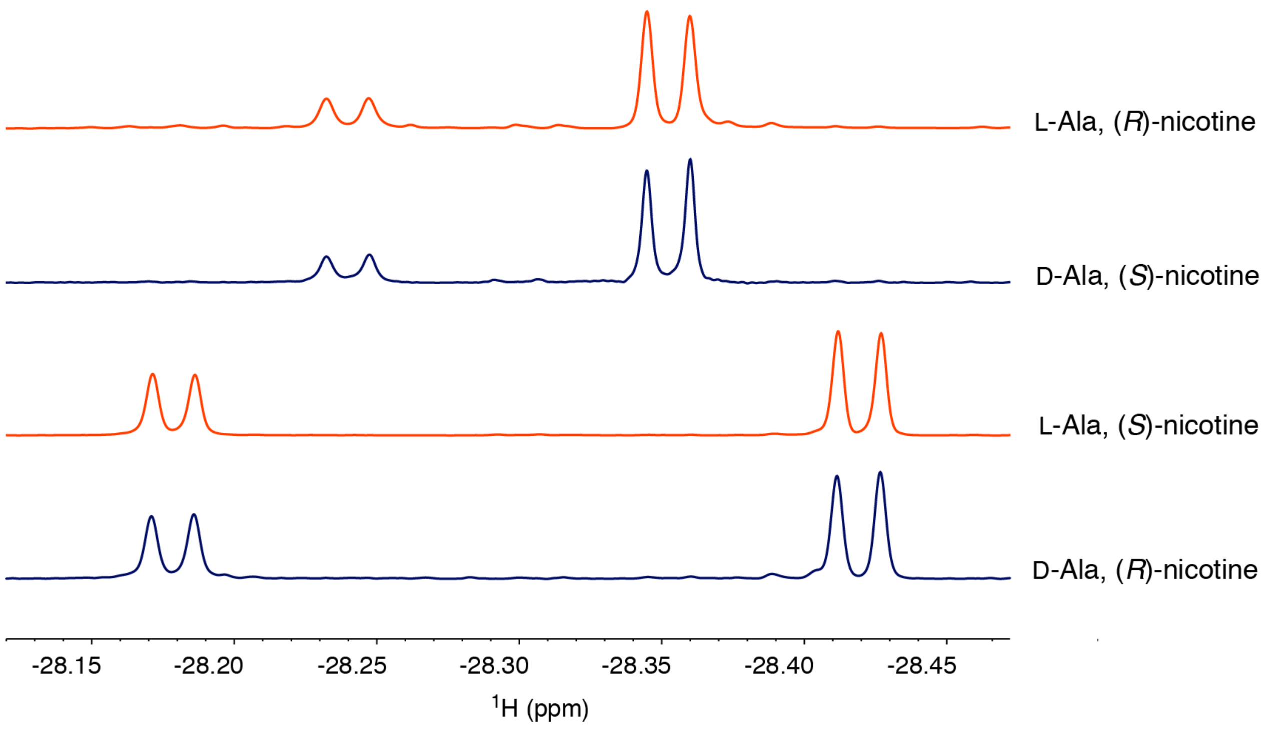

Figure S.3. nhPHIP-NMR signals of the high field hydrides for different homochiral alanine and nicotine ligands. All samples contained 100 μM alanine, 200 μM Ir-IMes catalyst, 3.6 mM nicotine and 5 bar of 51%-enriched *p*-H_2. Spectra were acquired at 10 °C and 600 MHz ^{1}H resonance frequency. The chirality of nicotine and alanine is indicated. Minor signals originate from sample impurities.

In summary, with 3 independent chiral centres, viz. the chelation at Ir, the amino acid, and the nicotine, one can expect as many as 2**3 = 8 stereoisomers, which are 4 pairs of enantiomers, so that 4 separate complexes are observable in the NMR spectrum.

S3 NMR experiments

All NMR experiments were performed at 278 or 283 K on an Agilent Unity INOVA spectrometer operating at 500 MHz ^{1}H resonance frequency or a Bruker AVANCEIII spectrometer operating at 600 MHz ^{1}H resonance frequency. Both spectrometers use a HCN triple-resonance cryo-cooled probe equipped with z-pulsed field gradients.

Hydride nhPHIP 1D NMR spectra were typically acquired with 16 or 32 transients in 1-3 minutes using a SEPP (selective excitation of polarization using PASADENA)[2,3] pulse scheme, centered approximately at -26 ppm.

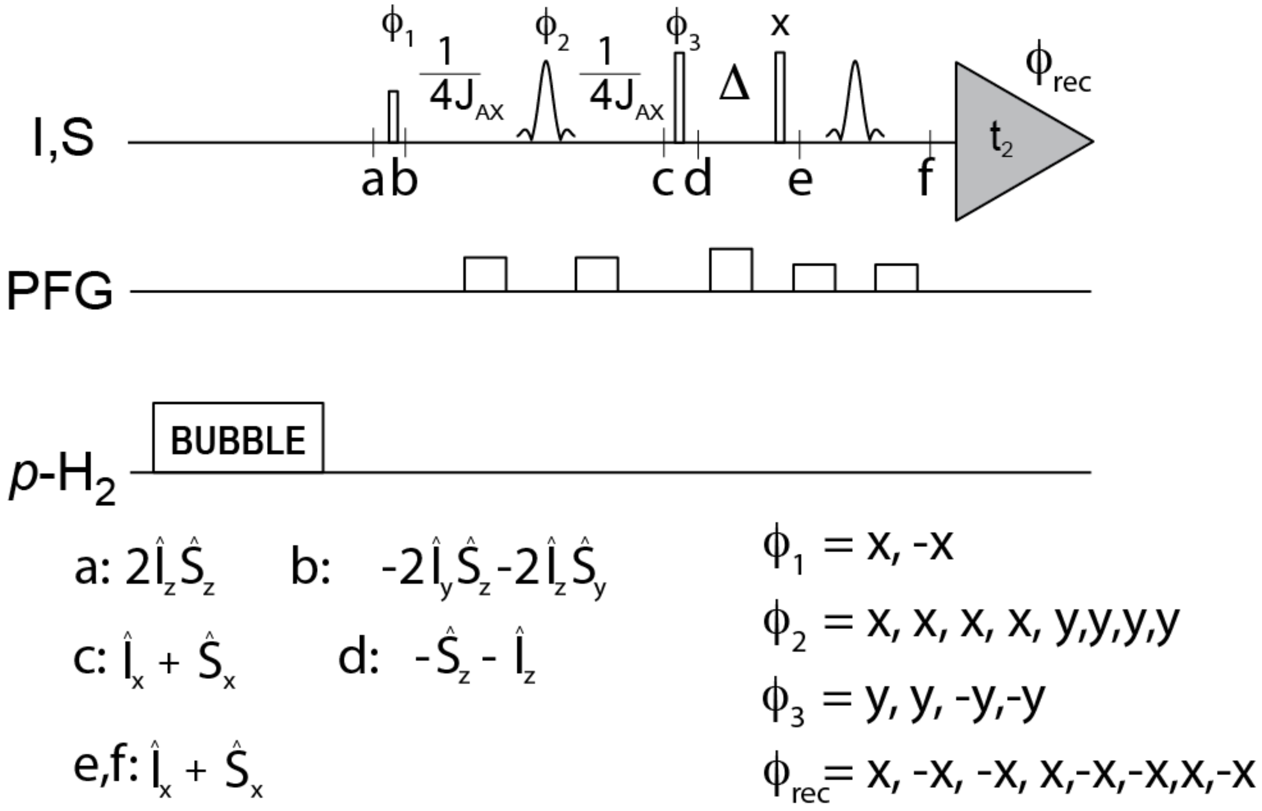

Figure S.4: A Pulse scheme to acquire a hydride 1D nhPHIP NMR spectrum. Phase cycling: ϕ_1: x, -x; ϕ_2: 2(y), 2(-y); ϕ_3: 4(x), 4(y), 4(-x), 4(-y); ϕ_4: 4(y), 4(-x), 4(-y), 4(x); ϕ_{rec}: x,-x,-x,x, y,-y,-y,y, -x,x,x,-x, -y,y,y,-y. The transmitter offset is set to ca. −26 ppm. Bubbling p-H_2 in the sample (between 0.5 and 3 s) occurs under spectrometer control at the beginning of each transient. The small and large rectangular pulses indicate 45° and 90° pulses, respectively, while shaped pulses represent selective *reburp* pulses with a bandwidth of 6000 Hz. J_{AX} denotes the average inter-hydrides scalar coupling constant (9 Hz). The acquisition time is indicated by t_2. Relevant terms of the density operator at different time points are indicated.

The pulse scheme of the 2D nhPHIP zero quantum NMR experiment is sketched in Figure S.5. After p-H_2 bubbling at the beginning of each transient, hydrides longitudinal spin order is converted to two-spin coherence via a 90° pulse. This is followed by a t_1

evolution period. A gradient dephases all coherence orders, except zero-quantum coherence. Thereafter, zero-quantum coherence is converted to antiphase, refocused and detected. 2D experiments were acquired with two transients per increment, using 1800 Hz spectral width in the indirect dimension (folding the signal twice). Typical size of the 2D datasets was 400(t_1, real)x3000(t_2, complex) or 600(t_1, real)x3000(t_2, complex) points.

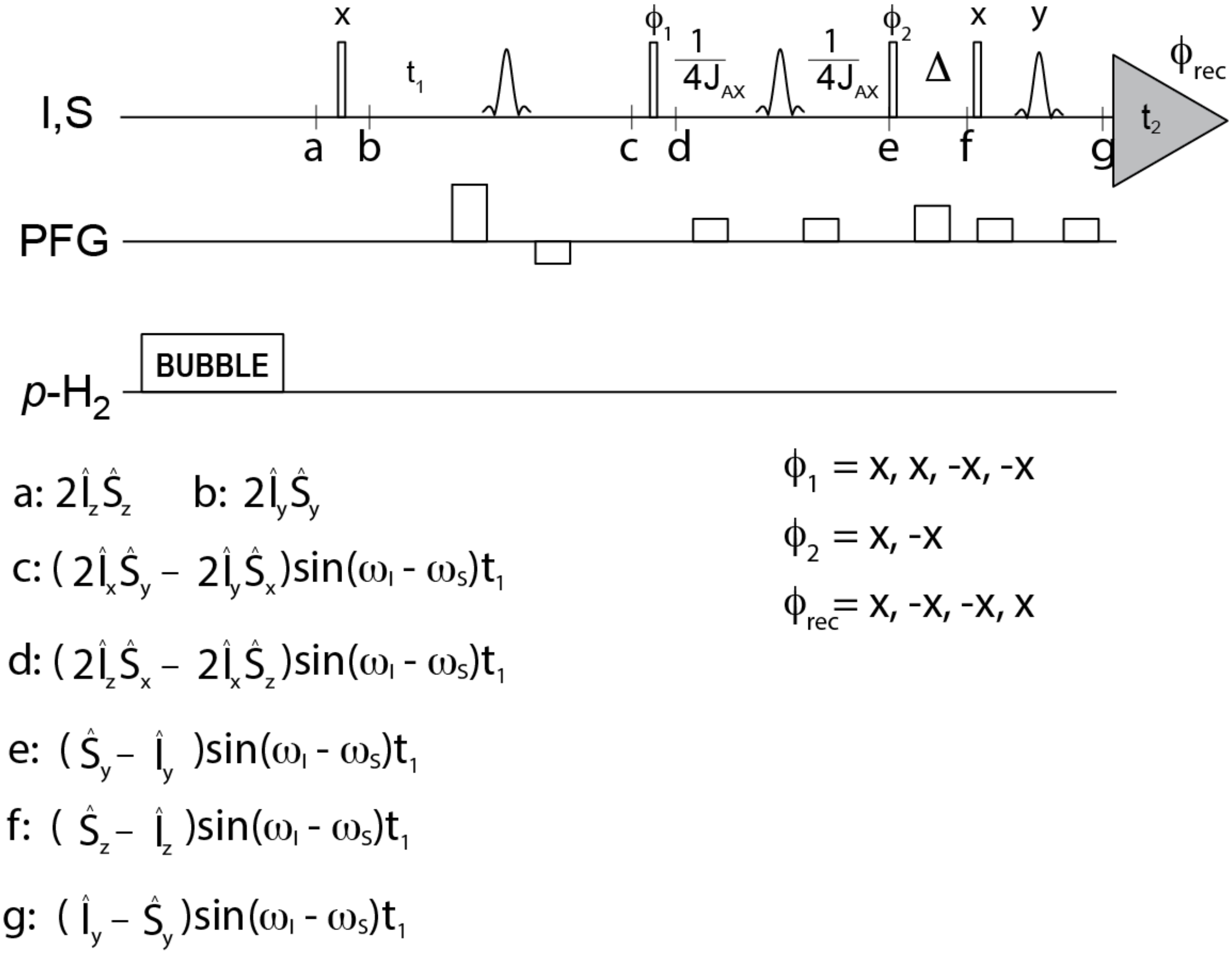

Figure S.5: Pulse scheme of 2D nhPHIP zero quantum experiment. Phase cycling follows: ϕ_1 = x, x, −x, −x; ϕ_2 = x, −x; ϕ_{rec} = x, −x, −x, x. The transmitter offset is to -25.76 ppm. p-H_2 bubbling (0.75 s or 1.5 s) is iterated under spectrometer control at the beginning of each transient. Hard 90° pulses are indicated by rectangular boxes. Shaped pulses represent selective *reburp* pulses with a bandwidth of 6000 Hz. J_{AX} indicates the average inter-hydrides scalar coupling constant (~9 Hz). The relevant spin density operator terms are indicated.

The 1D data sets were processed and the spectra were analyzed with iNMR (http://www.inmr.net). The 2D data sets were processed with NMRPipe[4] using Lorentz-to-Gauss apodization in t_2 and t_1, prior to zero filling to 4096(t_1, real)×32768(t_2, complex) points and Fourier transformation. NmrDraw and iNMR (http://www.inmr.net) were used

to analyze the 2D spectra. In order to determine the signals integrals, 2D peaks were fitted to Gaussian profiles in both the *F*1 and *F*2 dimensions by using one of the NMRPipe software tools, nlinLS for nonlinear least-squares minimization with five adjustable parameters (*F*2 position, *F*2 width, *F*1 position, *F*1 width, and amplitude). For the quantification of 1D signals, peaks were fitted using iNMR.

S4 Resonance Assignment of amino acids enantiomers

A CH_3OH/H_2O solution (19:1, 800 μL) containing the Ir-complex precursor (400 μM), (*S*)-nicotine (7.2 mM), piperidine/piperidinium buffer (1:1, 20 mM) and the racemate or L-enantiomer of the amino acid of interest (Xaa, 200 μM) was prepared by weighing. The solution (650 μL) was transferred into a 5 mm quick pressure valve (QPV) NMR tube (Wilmad-LabGlass). Thereafter, the QPV tube was sealed with an in-house-built headpiece to which three PEEK tube lines are connected. Nitrogen gas was passed through the solution to remove dissolved oxygen, after which the tube was pressurised under 5 bar of H_2. This resulted in the hydrogenation (activation) of the Ir-complex precursor upon which the activated symmetric complex [Ir(IMes)(H_2)(*S*-Nic)$_3$]Cl and the nhPHIP inactive, amino acid complex [Ir(IMes)(H_2)(Xaa)(*S*-Nic)]Cl were formed. The sample was subjected to heating at 50 °C for 7.5 min in a water bath to allow the formation of the nhPHIP active amino acid complex [Ir(IMes)(H_2)(Xaa)(*S*-Nic)]Cl.[5] The samples were measured at 10 °C (typically 16 transients) at 500 MHz, ^{1}H resonance frequency.

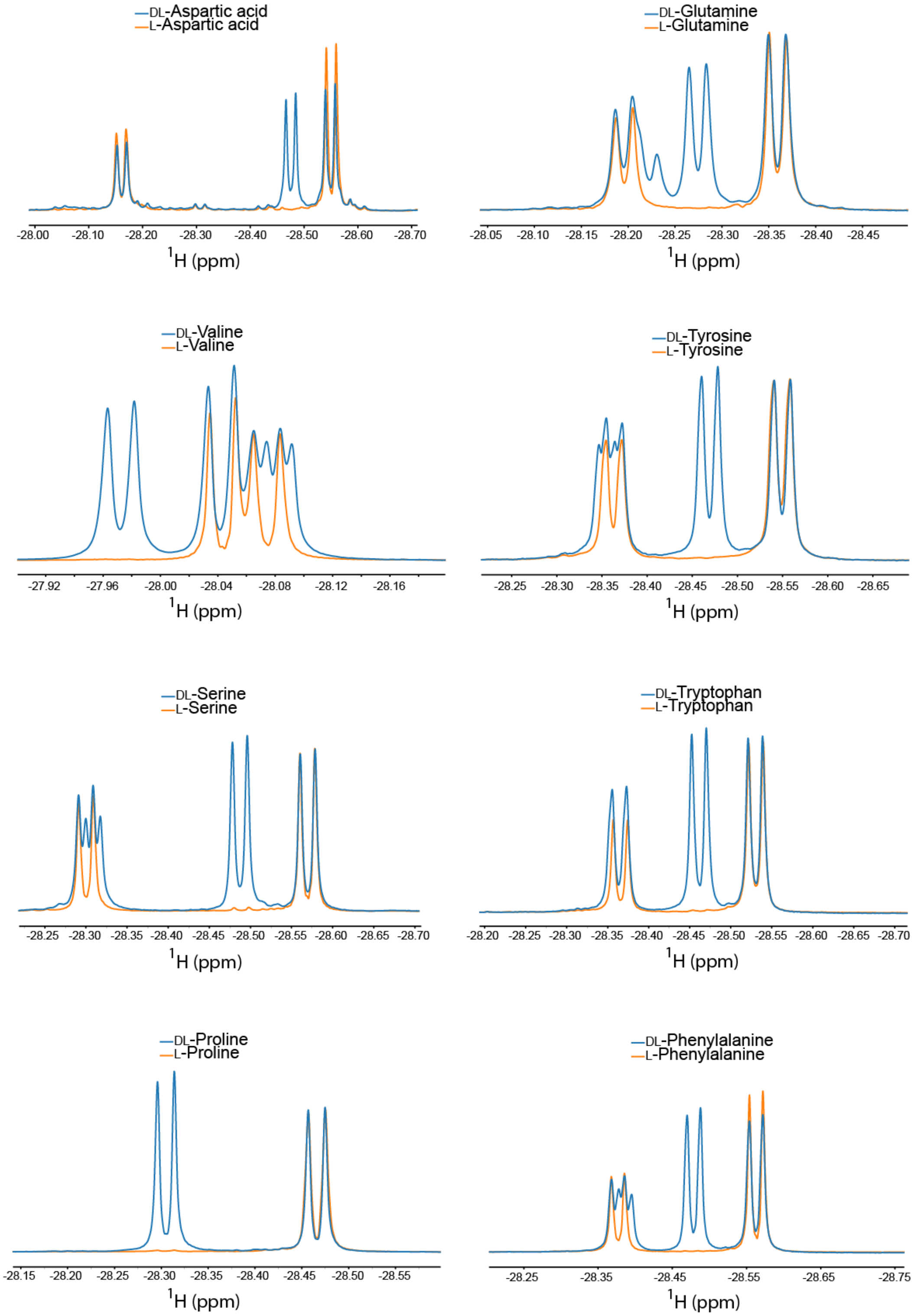

Figure S.6a: Superposition of the high-field region of 1D nhPHIP-NMR hydride spectra of the L-enantiomer (orange) and the racemic mixture (blue) for eight α-amino acids investigated in this study. Minor signals originate from sample impurities.

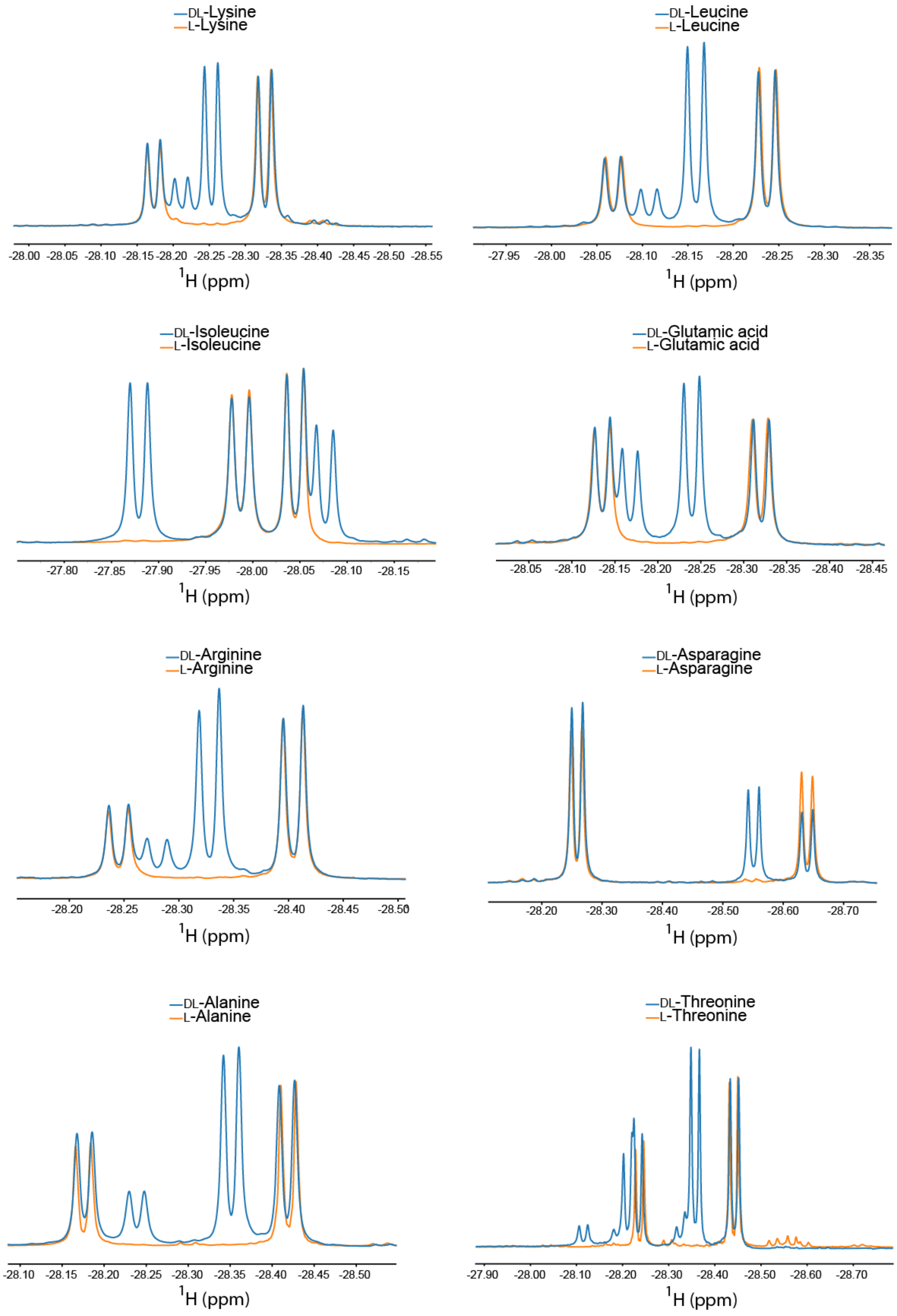

Figure S.6b: Superposition of the high-field region of 1D nhPHIP-NMR hydride spectra of the L- enantiomer (orange) and the racemic mixture (blue) for eight α-amino acids investigated in this study. Minor signals originate from sample impurities.

Table S.1 Chemical shifts of the hydrides complexes for glycine and the 16 DL-α-amino acids considered in this study measured at 10 °C in CH_3OH:H_2O 95:5 (v/v).

Amino acid	Low field hydride (ppm)	High field hydride (ppm)
L-Ala	-23.492	-28.419
	-23.568	-28.178
D-Ala	-23.510	-28.352
	-23.556	-28.240
L-Arg	-23.395	-28.404
	-23.566	-28.244
D-Arg	-23.434	-28.327
	-23.558	-28.279
L-Asn	-23.388	-28.639
	-23.526	-28.257
D-Asn	-23.419	-28.551
	-23.526	-28.257
L-Asp	-23.526	-28.544
	-23.555	-28.152
D-Asp	-23.454	-28.473
	-	-
L-Glu	-23.535	-28.312
	-23.598	-28.128
D-Glu	-23.564	-28.231
	-23.586	-28.161
L-Gln	-23.542	-28.361
	-23.601	-28.197
D-Gln	-23.571	-28.276
	-23.590	-28.224
Gly	-23.479	-28.599
	-23.494	-28.534
L-Ile	-23.704	-28.043
	-23.677	-27.984
D-Ile	-23.697	-28.075
	-23.690	-27.876
L-Leu	-23.583	-28.235
	-23.572	-28.066
D-Leu	-23.600	-28.157
	-23.554	-28.106
L-Lys	-23.513	-28.325
	-23.586	-28.170
D-Lys	-23.535	-28.250
	-23.573	-28.208

Amino acid	Low field hydride (ppm)	High field hydride (ppm)
L-Phe	-23.429	-28.564
	-23.659	-28.378
D-Phe	-23.459	-28.480
	-23.660	-28.387
L-Pro	-23.653	-28.465
	-	-
D-Pro	-23.695	-28.304
	-	-
L-Ser	-23.501	-28.570
	-23.594	-28.300
D-Ser	-23.516	-28.490
	-23.614	-28.310
L-Thr	-23.468	-28.443
	-23.650	-28.234
D-Thr	-23.487	-28.358
	-23.705	-28.211
L-Trp	-23.439	-28.527
	-23.778	-28.361
D-Trp	-23.461	-28.459
	-23.778	-28.361
L-Tyr	-23.475	-28.550
	-23.735	-28.364
D-Tyr	-23.502	-28.470
	-23.746	-28.355
L-Val	-23.624	-28.074
	-23.714	-28.041
D-Val	-23.722	-28.082
	-23.654	-27.972

S5 Artificial mixture of amino acids racemates

A CH_3OH/H_2O solution (19:1, 800 μL) containing the Ir-complex precursor (833 μM), (*S*)-nicotine (15 mM), piperidine/piperidinium buffer (1:1, 20 mM) and a racemic mixture of 16 proteinogenic α-amino acids (10 μM each enantiomer) and glycine (20 μM) was gravimetrically prepared. Deoxygenation and activation were conducted as described in the previous section. The spectrum was acquired at 10 °C in 50 minutes.

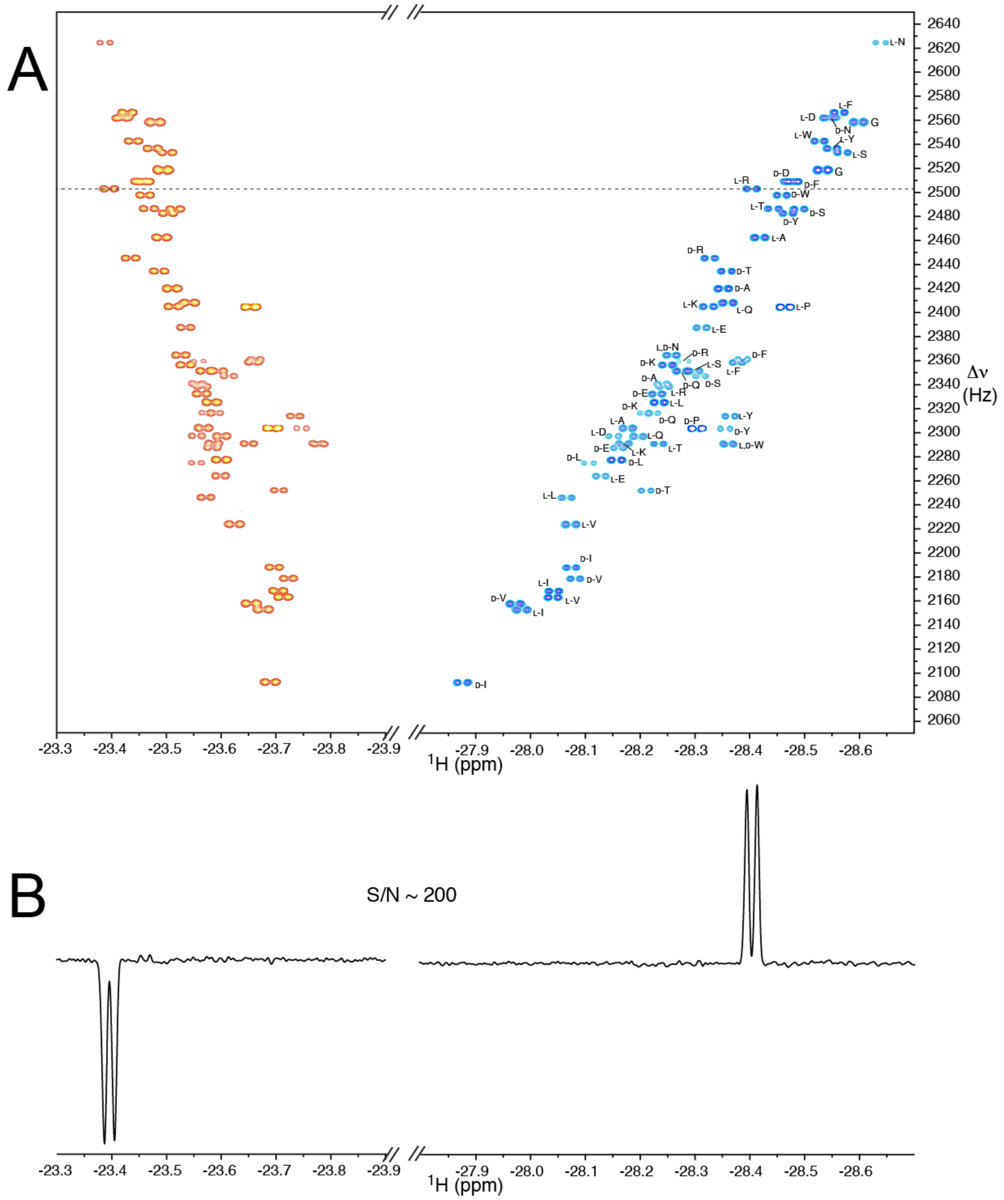

Figure S.7: A 2D nhPHIP zero quantum spectrum acquired at 500 MHz, ^{1}H resonance frequency for a racemic mixture of sixteen α amino acids and glycine. **B** 1D trace of the hydrides signals of L-Arg to illustrate the signal-to-noise ratio in the spectrum.

Signal assignment is reported in Figure S.7 using the one-letter code for amino acids. The correspondence between the full name, three-letter and one-letter code is given in table S.2.

Table S.2. Correspondence between name, three-letter and one-letter code for the amino acids considered in this study.

Name	3-letter	1-letter
Alanine	Ala	A
Arginine	Arg	R
Asparagine	Asn	N
Aspartic Acid	Asp	D
Glutamic Acid	Glu	E
Glutamine	Gln	Q
Glycine	Gly	G
Isoleucine	Ile	I
Leucine	Leu	L
Lysine	Lys	K
Phenylalanine	Phe	F
Proline	Pro	P
Serine	Ser	S
Threonine	Thr	T
Tryptophane	Trp	W
Tyrosine	Tyr	Y
Valine	Val	V

S6 Enantiomeric ratio from nhPHIP-NMR

The integrals of the hydride signals for the D- and L- amino acid complexes can provide a direct estimate of the enantiomeric ratios in solution, provided differences in nhPHIP efficiencies are accounted for. This can be accomplished by measuring the L-/D- hydride integrals ratio (R_{rac}) for a racemic mixture, choosing the most resolved and/or intense L- and D- hydride signals in the nhPHIP-NMR spectrum. In the example shown in figure s.8 (top) a value of R_{rac}=1.02 was determined for a racemic solution of alanine (E_r=1). An integral ratio equal to 1.98 was measured for a second sample with enantiomeric ratio equal to 1.96 (bottom), in good agreement with the value obtained from the expression:

$$E_r = \frac{C_L}{C_D} = \frac{1}{R_{rac}} \times \frac{\text{Integral}(L)}{\text{Integral}(D)} = \frac{R}{R_{rac}} = \frac{1.98}{1.02} = 1.95$$

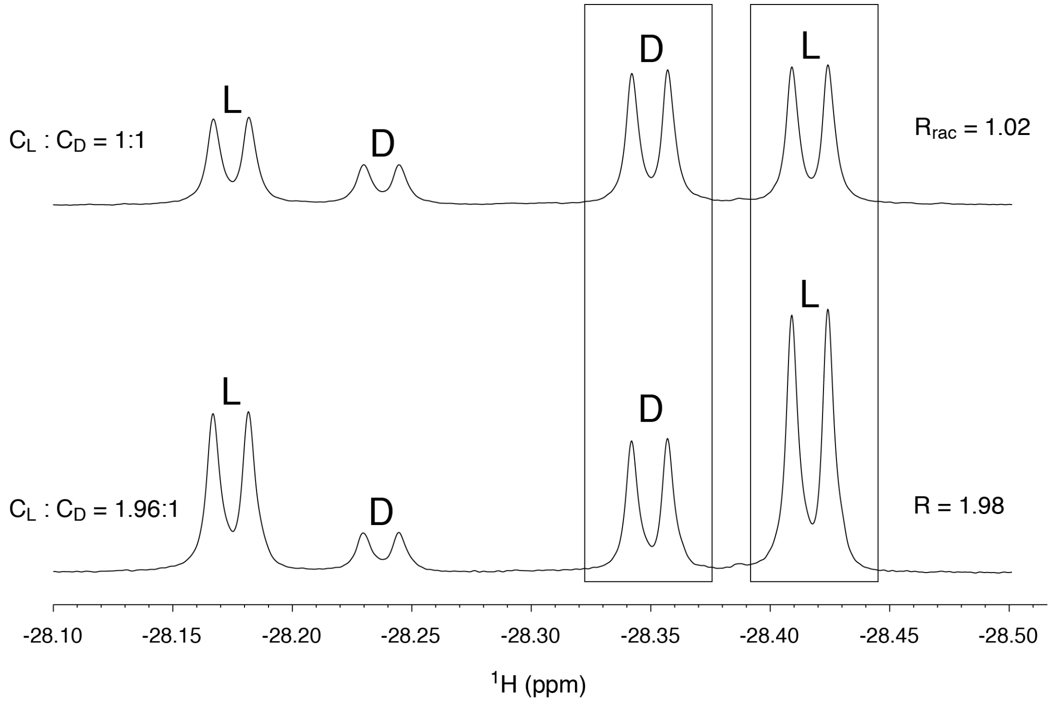

Figure S.8: 1D nhPHIP-NMR spectra of a 20 μM racemic solution of alanine (Er = 1) (**Top**) and a 60 μM solution of alanine with enantiomeric ratio Er = 1.96 (**Bottom**). The spectra were recorded at 600 MHz ^{1}H resonance frequency and 10 °C in CH_3OH:H_2O 95:5 (v/v), in the presence of 250 μM Ir-IMes catalyst, 4.5 mM (*S*)-nicotine, piperidine/piperidinium buffer (1:1, 10 mM) and 5 bar of 51%-enriched *p*-H_2. The amino acid solutions were prepared by diluting stock solutions with concentrations accurately determined via thermal NMR measurements.

S7 Integral ratio for enantiomers in different matrices

The NMR signal enhancement obtained via nhPHIP is generally influenced by the experimental conditions as well as by the solution composition. Therefore, the integral ratio between the nhPHIP signals of two different substrates does not necessarily remains constant as the solution composition changes. However, in the case of amino acids enantiomers, we have observed that the integral ratio is rather insensitive to changes in the matrix composition. Therefore, the value of the L/D integral ratio for a racemate, which is necessary for a quantitative analysis, can be measured on standard solutions even if they differ substantially from the mixture under investigation, provided experimental conditions such as magnetic field strength, solvent composition, temperature and pH are the same. In the following, we show that the L/D integral ratio for two amino acid racemates (i.e. isoleucine and pipecolinic acid (2-piperidine carboxylic acid), a non-proteionogenic amino acid) is the same within experimental error for two vastly different matrices such as methanol and a mixture of urine in methanol, as typically employed in amino acid measurements using nhPHIP. We have chosen urine for this comparison because of its complexity, the high concentration of salts and of ligands that might potentially interfere with a nhPHIP measurement on amino acids. The choice of isoleucine and pipecolinic acid is related to the fact that they resonate outside the most crowded spectral areas of urine nhPHIP spectra (see Figure S.9 below) and, therefore, they can be quantitatively determined using a simple 1D approach.

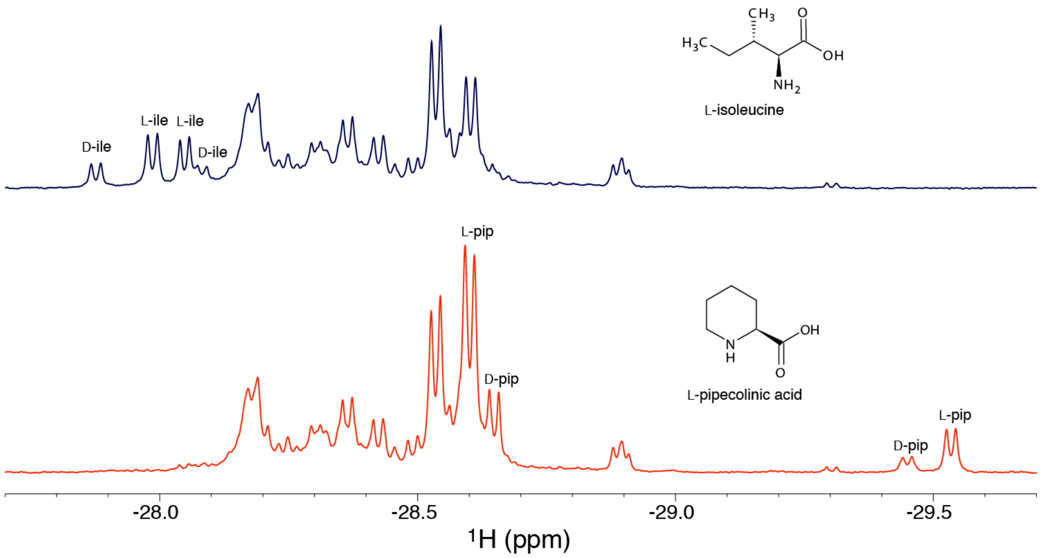

Figure S.9: 1D nhPHIP-NMR high-field hydride signals measured for urine spiked with (A) isoleucine D:L 1:2 and (B) pipecolinic acid D:L 1:2. Both spectra were acquired in CH_3OH:urine:H_2O 95:4:1. Enantiomer assignment is indicated. The structures of the L-enantiomers of the two amino acids are shown.

Importantly, these amino acids are not observed in the nhPHIP NMR spectra of unspiked urine. This allows preparing solutions of these amino acids in urine at concentrations accurately determined by gravimetry.

For each racemate two samples were prepared, one in $CH_3OH:H_2O$ 95:5 (v/v), the other in CH_3OH:urine:H_2O 95:4:1 (v/v). All samples contained Imes (833 μM), (*S*)-nicotine (15.0 mM) and piperidine/piperidinium buffer (1:1, 20 mM). The water content in all samples was adjusted to 5.0 % (v/v). Note that while water content and pH are virtually identical in the two mixed solvents, a much higher ionic strength is expected for the urine-methanol mixture. For isoleucine, the concentration in both samples was 49 μM per enantiomer. In the case of pipecolinic acid the concentration in the methanol sample was 36.4 μM for each enantiomer, while in the urine sample it was 38 μM per enantiomer. For all samples 1D nhPHIP hydrides spectra were measured under the same conditions at T = 5 °C. For both racemates, the integral ratio (R_{rac}) was determined for hydride signals free from the urine background by 1D fitting of the peaks. As illustrated in figure S.10, the L/D integral ratio is highly similar in the two matrices for both racemates.

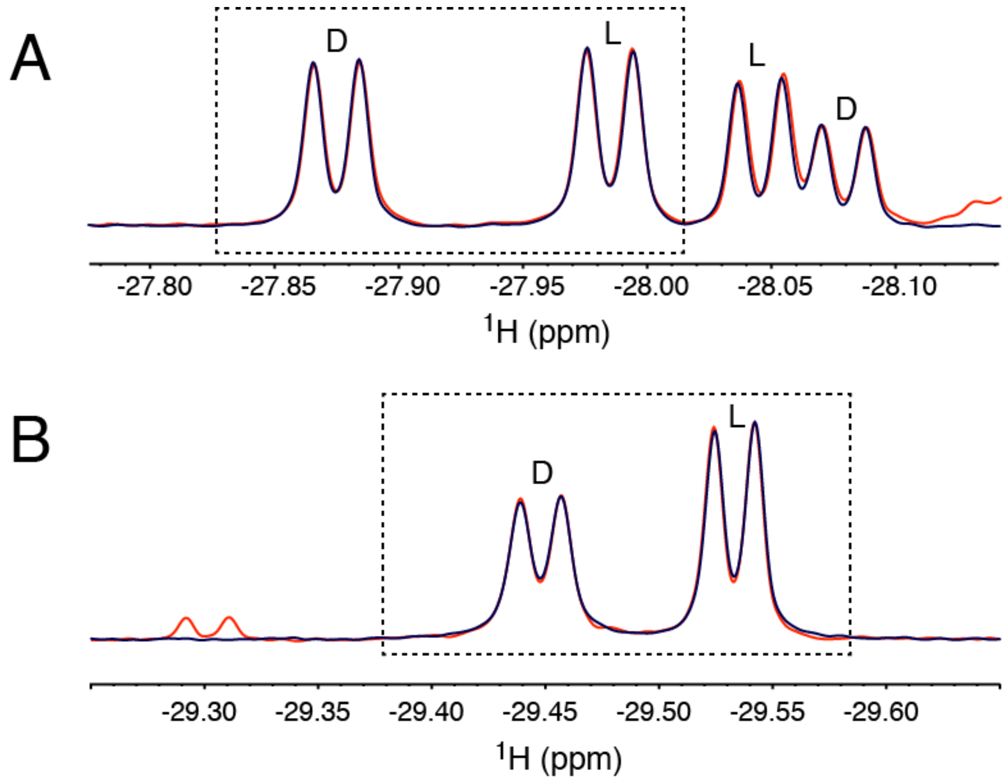

Figure S.10: 1D nhPHIP-NMR signals for the racemate of (A) isoleucine and (B) pipecolinic acid, measured in $CH_3OH:H_2O$ 95:5 (v/v) (blue line) and in CH_3OH:urine:H_2O 95:4:1 (red line). The hydride signals in the dashed boxes were used to calculate the integral ratio. Enantiomer assignment is indicated.

The results of the 1D peak fitting are summarized in Table S.3 below, displaying the agreement between the measurements in the two solutions.

Table S.3. L/D Integral ratio of the racemates of isoleucine and pipecolinic acid in two different matrices (indicated).

DL racemate	R_{rac} ($CH_3OH:H_2O$)	R_{rac} ($CH_3OH:urine:H_2O$)	SN ratio
isoleucine	1.09	1.09	300:1
pipecolinic acid	1.24	1.24	300:1

S8 Application to complex mixtures of known concentrations

Eight solutions of isoleucine or pipecolinic acids were gravimetrically prepared at four different enantiomeric ratios (e.g L:D = 1:1.5, 1:2, 1:2.5, 1.3) using a mixture CH_3OH:urine:H_2O 95:4:1 (v/v) as a solvent. The nhPHIP method was used to determine the enantiomeric ratios for both amino acids and the results were compared to the reference values obtained gravimetrically. In addition, the enantiomer concentrations were determined for both amino acids in the mixtures with enantiomeric ratio 1:2.5. All solutions contained Imes (833 μM), (*S*)-nicotine (15.0 mM), piperidine/piperidinium buffer (1:1, 20 mM). The water content in all samples was adjusted to 5.0 % (v/v). Table S.4 details the composition of the eight samples, together with the quantification of the enantiomeric ratios via the nhPHIP approach.

Table S.4. Comparison between enantiomeric ratios determined gravimetrically or via the nhPHIP method for eight solutions of isoleucine or pipecolinic acid in a CH_3OH/urine mixture.

Sample	Amino acid	L_{grav} (μM)	D_{grav} (μM)	E_r^{grav}	L_{exp} (μM)	D_{exp} (μM)	I_r	E_r^{exp}	deviation
A	Ile	30.2	15.1	2.00			2.19	2.01	+0.5%
B	Ile	35.0	14.0	2.50	35.8 (+2%)	14.3 (+2%)	2.72	2.50	-
C	Ile	45.8	15.3	2.99			3.31	3.03	+1%
D	Ile	35.0+27.8	14.0+27.8	1.50			1.65	1.51	+0.7%
E	Pip. acid	32.6	16.3	2.00			2.56	2.07	+4%
F	Pip. acid	35.0	14.0	2.50	35.0 (-)	14.0 (-)	3.10	2.50	-
G	Pip. acid	45.8	15.2	2.99			3.60	2.90	-3%
H	Pip. acid	35.0+28.0	14.0+28.0	1.50			1.85	1.50	-

In Table S.4 L_{grav} and D_{grav} indicate the concentrations of the two enantiomers determined gravimetrically and E_r^{grav} indicates the corresponding enantiomeric ratio. The enantiomer concentrations determined via nhPHIP are indicated by L_{exp} and D_{exp}, while I_r and E_r^{exp} are the integral ratio and the enantiomeric ratio obtained via nhPHIP. The last column reports the percent deviation of the enantiomeric ratios determined via nhPHIP from the gravimetrically determined one.

For the quantitative determination of the concentrations of the isoleucine enantiomers in the solution with enantiomeric ratio equal to 2.5 the procedure outlined in the main text was followed:

1) determination of the enantiomeric ratio of solution B via nhPHIP
2) determination of the enantiomeric ratio via nhPHIP of solution D, obtained by spiking solution B with a known concentration of racemic isoleucine.

Identical procedure was followed for solutions F and H in the case of pipecolinic acid.

As discussed in the main text, the enantiomer concentrations can be calculated from:

$$C_D = \frac{E_r^{spiked} - 1}{E_r - E_r^{spiked}} \times C_D^{spiked}$$

$$C_L = E_r \times C_D$$

As reported in Table S.4, deviations in the order of +/- 2% were observed for the enantiomer concentrations for both amino acids.

S9 Instant coffee

Instant coffee of the brand "NESCAFÉ GOLD" (1g) was suspended in CH_3OH (5g). After 30 min on a sample rotator, the sample was centrifuged for 3 minutes via a manual centrifuge and the supernatant was stored in a separate vial. A CH_3OH/H_2O reference solution (19:1, 800 μL) containing the Ir-complex precursor (833 μM), (*S*)-nicotine (15 mM), piperidine/piperidinium buffer (1:1, 20 mM) and the coffee extract (10 vol%) was prepared by weighing. Two CH_3OH/H_2O spiking solutions (19:1, 800 μL) containing the Ir-complex precursor (833 μM), (*S*)-nicotine (15 mM), piperidine/piperidinium buffer (1:1, 20 mM), two amino acid racemates of interest (DL-Val 5.1 μM, DL-Ile 5.1 μM) and the coffee extract (10 vol%) was prepared by weighing. Deoxygenation and activation were conducted as

described in the previous sections. The experiments were acquired at 500 MHz, ^{1}H resonance frequency. Each spectrum was recorded in 1 hour.

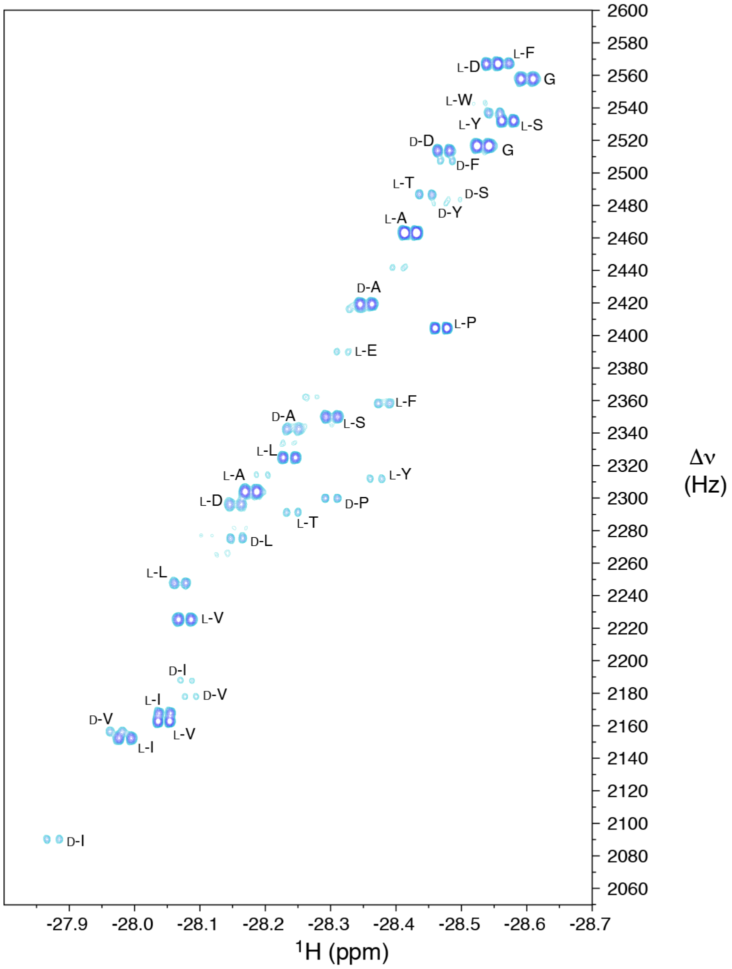

Figure S.11: High-field region of the 2D nhPHIP zero quantum spectrum acquired on coffee extract at 5 °C in CH_3OH:H_2O 95:5 (v/v). Assignments of the amino acids enantiomers are indicated.

S10 Enantiomeric ratio of Ala and Ser in human urine

A urine sample was collected from a healthy volunteer. Informed consent was obtained for the experiment on this sample to be included in the article. All procedures were in accordance with the Helsinki Declaration of 1975, as revised in 2000. Approval for the study was obtained from the Research Ethics Committee of the Faculty of Science of the Radboud University (Approval number: REC21111). The urine sample was collected using routine clinical collection protocols and stored at −80 °C. A CH_3OH/H_2O reference solution (19:1, 800 μL) containing the Ir-complex precursor (833 μM), (*S*)-nicotine (15 mM), piperidine/piperidinium buffer (1:1, 20 mM) and urine (5 vol%) was prepared by weighing. Beforehand, urine was mixed with piperidine/piperidinium buffer stock

solution. Before adding, the urine/buffer solution was centrifuged (13500 rpm, 3 min) in order to remove potential precipitate. Deoxygenation and activation were conducted as described in the previous sections. The spectrum was acquired at 500 MHz, ^{1}H resonance frequency in 33 minutes.

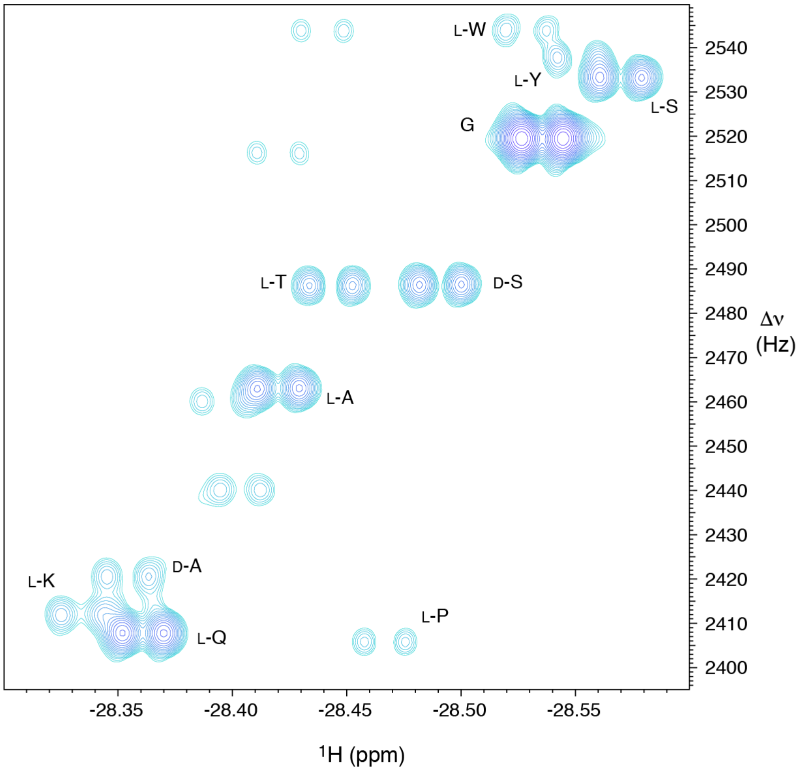

Figure S.12: Portion of the high-field region of the 2D nhPHIP Zero Quantum spectrum displaying the hydride resonances used to determine the enantiomeric ratios for alanine and serine in human urine. The experiment was acquired on a sample CH_3OH:urine 95:5 (v/v) at 10 °C. Assignments of the amino acids enantiomers for the main signals are indicated. Unassigned signals derive presumably from urine metabolites carrying α- amino acid functional groups.

S11 References

(1) Kelly III, R. A.; Clavier, H.; Giudice, S.; Scott, N. M.; Stevens, E. D.; Bordner, J.; Samardjiev, I.; Hoff, C. D.; Cavallo, L.; Nolan, S. P. *Organometallics* **2007**, *27*, 202.

(2) Sengstschmid, H.; Freeman, R.; Barkemeyer, J.; Bargon, J. *J. Magn. Reson., Ser. A* **1996**, *120*, 249–257.

(3) Barkemeyer, J.; Bargon, J.; Sengstschmid, H.; Freeman, R. *J. Magn. Reson., Ser. A* **1996**, *120*, 129–132.

(4) Delaglio, F.; Grzesiek, S.; Vuister, G. W.; Zhu, G.; Pfeifer, J.; Bax, A. *J. Biol. NMR* **1995**, *6*, 277-293.

(5) Sellies, L.; Aspers, R. L. E. G. ; Feiters, M. C.; Rutjes F. P. J. T.; Tessari M. *Angew. Chem. Int. Ed.* **2021**, *60,* 26954 – 26959.
